# Lipids and lipid nanoparticles functionalized with randomized poly(ethylene glycol) (rPEG) for mRNA delivery

**DOI:** 10.1039/d6sc00169f

**Published:** 2026-05-29

**Authors:** Philip Dreier, Caroline Bockhard, David Seibel, Olaf Soltwedel, Rebecca Matthes, Gregor M. Linden, Julian Schmidt, Dominik Göbel, Thomas Endres, Johannes Scheiger, Regine von Klitzing, Emanuel Schneck, Holger Frey

**Affiliations:** a Department of Chemistry, Johannes Gutenberg-University Mainz Duesbergweg 10-14 55128 Mainz Germany hfrey@uni-mainz.de; b Evonik Operations GmbH Kirschenallee 64293 Darmstadt Germany; c Evonik Operations GmbH Rodenbacher Chaussee 4 63457 Hanau Germany; d Institute for Condensed Matter Physics, Technische Universität Darmstadt Hochschulstrasse 8 64289 Darmstadt Germany

## Abstract

PEG lipids are essential excipients in lipid nanoparticle (LNP) formulations and enable the transport of therapeutic ribonucleic acids, creating stealth properties for these nanocarriers. However, an increasing number of studies raise concerns related to the growing presence of anti-PEG antibodies (APA) in the population. The presence of APAs results in the recognition and accelerated blood clearance of PEGylated therapeutics, thus diminishing the desired stealth effect of PEGylation. In this study, we present the isomerization of PEG to randomized PEG (rPEG) as an efficient approach for inhibiting APA interaction while preserving the advantages of PEG. rPEGs were obtained *via* living anionic ring-opening (co)polymerization (AROP) of ethylene oxide (EO) with glycidyl methyl ether (GME). This yields rPEGs with narrow molar mass distributions (*M*_w_/*M*_n_ = 1.04–1.10) for all EO:GME compositions synthesized. Chain-end functionalization of rPEGs with lipid anchor groups was conducted to obtain rPEG lipids with C_14_-chains (ditetradecylacetamide (DTAA), dimyristoyl-glycerol (DMG)). APA interaction was investigated *via* enzyme-linked immunosorbent assay (ELISA) and X-ray reflectometry (XRR) at monolayers of the rPEG lipid, allowing for a general understanding of the relation between the chain architecture and APA interaction. LNPs containing rPEG lipids were formulated and compared to established PEG-based LNPs, showing similar particle sizes, encapsulation and transfection efficiencies, and cell viabilities for a variety of cell lines. It is demonstrated that rPEG lipids can prevent APA binding but nevertheless exhibit similar performance in LNPs as comparable PEG lipids.

## Abstract 2

rPEG lipids have been prepared as novel excipients for lipid nanoparticle (LNP) formulations, enabling the transport of therapeutic ribonucleic acids.

## Graphical abstract

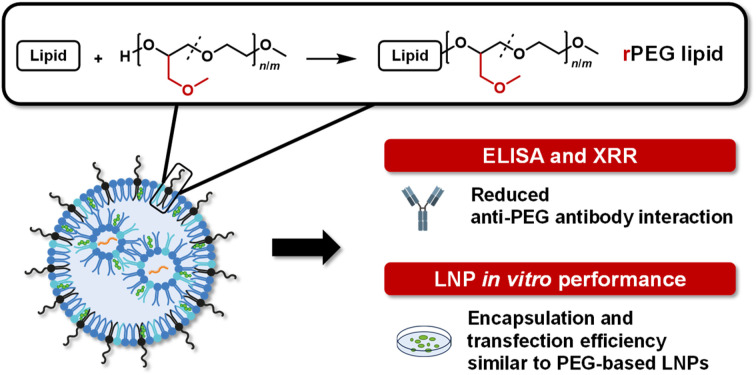

## Introduction

The emergence of messenger RNA-based vaccines (mRNA), such as those developed for COVID-19, represents a groundbreaking advancement in the field of nanomedicine and vaccinology.^1^ These vaccines harness the inherent property of mRNA to instruct cells to produce specific viral proteins, triggering an immune response. One critical aspect that has played a pivotal role in the success of these vaccines is their delivery system: lipid nanoparticles (LNP).^2,3^ Such LNPs consist of four types of lipids, one of which is a poly(ethylene glycol) (PEG) attached to a lipid anchor, commonly referred to as PEG lipid.^4^ Together with (i) stabilizing cholesterol, (ii) an ionizable amino lipid complexing the negatively charged mRNA and (iii) “helper” phospholipids facilitating a lipid bilayer, PEG lipids are formulated to LNPs relying on microflow techniques, enabling the controlled transport of mRNA.^5^ In this context, PEGylation leads to the particular “stealth effect”,^6,7^ which allows for enhanced stability of LNPs regarding nuclease-induced degradation as well as long-term stability during storage and delivery. This is particularly crucial to maintain the integrity of the mRNA vaccine until it reaches its desired destination in the body. The PEG lipid enables precise control of the particle size and prevents aggregation during storage as it acts as a steric barrier.^8^ Especially in regard to LNP formulation and lipid synthesis, PEG offers an unmatched versatility of applicable organic solvents, ranging from water to aromatic solvents like toluene.^9^ The onset of the COVID-19 pandemic has amplified the impact of anti-PEG antibodies (APA). Prior to the COVID-19 pandemic, the percentage of individuals with APAs was already at 83% in western countries.^10^ The presence of APAs in the bloodstream reportedly leads to an accelerated blood clearance, hypersensitivities,^11,12^ and strong allergic reactions up to anaphylactic shock^13^ against PEGylated drugs and liposomal formulations such as Doxil^®^. APAs are evident in individuals who have received prior treatment with PEGylated drugs as well as in those who have never been treated with PEGylated pharmaceuticals.^14,15^ The extensive usage of PEG in cosmetics and food additives is held accountable for this phenomenon. The unprecedented and nearly simultaneous introduction of PEGylated lipids on a global scale marks a groundbreaking situation in the context of APAs. Vaccination with Comirnaty® (BioNTech/Pfizer) and Spikevax® (Moderna)^5^ have led to a significant increase of IgG (1.8-fold and 13.1-fold) and IgM (2.6-fold and 68.5-fold), respectively.^16^ Previous research elucidated the recognition mechanism of PEG by APAs. APAs can be categorized into backbone- and end group-specific antibodies. In the case of both types, the interaction between the PEG backbone and the antibody paratope is facilitated through van der Waals and hydrogen bond interactions. The Fab paratope of a recently investigated backbone-specific APA binds to at least 16 EO units of PEG.^17^ In comparison, the end group-specific APA not only captures the methoxy moiety of mPEG but also interacts with several terminal ethylene oxide (EO) units.^18^ Although APAs are labelled as “specific”, *e.g.*, end group-specific APAs also bind, with a lower affinity, to the backbone. Thus, they feature end group selectivity rather than true specificity.^19,20^ The binding of antibodies to PEG chains mimicking the surfaces of PEGylated foreign bodies or liposomes has previously been structurally characterized with neutron reflectometry (NR), evidencing the formation of dense antibody layers even at moderate PEG grafting densities.^21,22^

Over the past two decades, various potential substitutes for PEG have been designed,^23,24^ which aim to preserve PEG's unique property profile: high water solubility, excellent biocompatibility, and an overall high chemical stability. A subset of these alternatives has already been utilized in the synthesis of PEG-like lipids and the corresponding LNPs. Among others,^25^ polysarcosine,^26,27^ polyoxazolines^28^ and poly(oligo(ethylene glycol)methacrylate) (POEGMA)^29^ have been developed for lipids as well as bioconjugation and studied by numerous groups. Each of these alternatives requires customized synthesis routes as well as individualized polymer-lipid conjugation chemistry. Due to the challenges posed by the COVID-19 pandemic, the biopharmaceutical industry witnessed accelerated product development, clinical development, and shortened authority approval times.^30,31^ This was particularly notable in the case of lipid manufacturing, where established and scalable production and GMP protocols play a crucial role. Efficient integration of new technology into existing value chains in terms of cost and time is a crucial determinant for its practical implementation. Here, we present the synthesis of polymer lipids and their incorporation in LNP formulations, which is based on constitutional isomers of linear PEG.^32^ The polyether-based PEG alternative is obtained by random anionic (co)polymerization of EO and its isomeric dimer glycidyl methyl ether (GME), resulting in randomized PEG (rPEG). The applied polymerization method, anionic ring-opening polymerization (AROP) aligns with the established industrial process to produce pharma-grade PEG, merely adding GME as an additional comonomer. The rPEG structure preserves the core properties of PEG: water solubility, biocompatibility, non-toxicity, as well as equal chemistry in terms of solubility, polymer synthesis, and post-modification. Yet most importantly, rPEGs, in contrast to PEG, are not antigens to APAs.^32,33^ The introduction of randomly distributed methoxy methylene side chains prevents recognition of the polyether backbone. This study showcases the tethering of rPEG to two clinically relevant lipid anchors synthesized *via* commercially viable pathways. The rPEG lipids are further utilized in LNP formulations, highlighting rPEGs' role as a PEG alternative.

## Results and discussion

### Synthesis of rPEG lipids

In this study, rPEG lipids were synthesized in analogy to the established synthesis routes of the commercially available lipids ALC-0159 (mPEG-*N*,*N*-ditetradecylacetamide (mPEG-DTAA)) and mPEG-1,2-dimyristoyl-glycerol (mPEG-DMG) utilized in the COVID-19 vaccines Comirnaty® (BioNTech/Pfizer) and Spikevax® (Moderna), respectively.^31^ Both lipid formulations rely on PEG lipids based on mPEG with a molar mass (*M*_n_) of 2.0 kg mol^−1^ (degree of polymerization (DP) = 45), ensuring the optimal hydrophilic/lipophilic balance essential for the formation of stable and well-defined LNPs. The copolymerization of EO and GME introduces methoxy methylene side chains to the polyether backbone, which potentially impacts the hydrodynamic radius of rPEG in comparison to PEG.^32^ Therefore, the design of rPEGs in the scope of this paper was motivated by the emulation of the hydrodynamic radius of PEG. Series of samples varying in their composition were synthesized in accordance with mPEG 2000, aiming either at the same molar mass (2.0 kg mol^−1^) or degree of polymerization (DP) (45) to determine paramount parameters, *i.e.*, molar mass and chain length, respectively, and their influence on cell viability, physicochemical and antigenic properties.

**Fig. 1:**
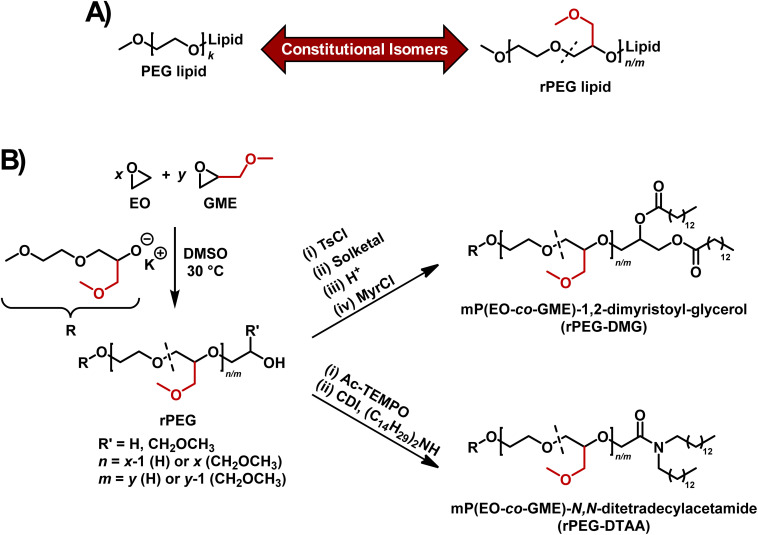
General concept and synthesis of rPEG lipids. (A) Structural comparison of PEG and rPEG; (B) synthesis procedure for rPEG lipids.

The rPEGs were synthesized *via* anionic ring-opening (co)polymerization of EO and GME in dimethyl sulfoxide (DMSO) at 30 °C (Fig. 1, Tables 1 and S1).^32^ GME was utilized as a racemic mixture, hence consecutive EO units are interrupted by either one of the two enantiomers of GME. Polymers ranging from 2.0 to 2.7 kg mol^−1^ were synthesized with low dispersities (*Đ*) ≤ 1.10 (Table S1).

**Table 1:** Overview of characterization of synthesized PEG and respective rPEG lipids

Sample	DP_calc._	DP_MALDI+NMR_^a^	GME_calc_ [%]	GME_NMR_^a^ [%]	*M*_n,calc._ [kg mol^−1^]	*M*_n,MALDI_ [kg mol^−1^]	*M*_n,SEC_ [kg mol^−1^]	*Đ*_SEC_
mPEG_45_-DTAA	45	45	0	0	2.5	2.6	2.4	1.02
rPEG^0.36^_33_-DTAA	33	33	36	36	2.4	2.3	1.9	1.04
rPEG^0.49^_30_-DTAA	30	30	50	49	2.4	2.3	1.9	1.04
rPEG^0.36^_43_-DTAA	44	43	36	36	3.0	2.8	2.4	1.06
rPEG^0.49^_41_-DTAA	44	41	50	49	3.1	3.0	2.5	1.07
mPEG_45_-DMG	45	45	0	0	2.5	2.3	2.3	1.04
rPEG^0.36^_45_-DMG	45	45	36	36	3.2	3.1	2.8	1.06
rPEG^0.48^_44_-DMG	44	44	50	48	3.5	3.4	2.9	1.04

a Determined from rPEG precursor.

Further, rPEGs with moderate (36 mol%) and large amounts (50 mol%) of incorporated GME units were targeted. Previous studies and simulations revealed that the introduction of GME decreases the statistical abundance of segments consisting of multiple consecutive EO repeating units in a chain,^32^ assuming an ethylene glycol sequence length of 16 units as the PEG epitope.^17^ The exploration of varying ratios of GME enables the assessment of antigenicity, LNP formulation, and mRNA transfection efficiency in this study. Subsequently, two types of lipids, rPEG-DMG and rPEG-DTAA, were synthesized based on industrially established synthesis routes. rPEG-DTAA was obtained *via* a two-step pathway by (i) oxidation of the terminal hydroxyl moiety of rPEG with (4-acetamido-2,2,6,6-tetramethylpiperidin-1-yl)oxyl (Ac-TEMPO) and (ii) subsequent amide formation of rPEG-acetic acid with carbonyl diimidazole and ditetradecylamine (Fig. 1B).

A lower yield for the synthesized lipids compared to PEG was observed, which is explained by the occurrence of secondary, terminal hydroxy units of 36 and 50 mol% in correspondence with the respective GME ratio in rPEG.^32^ The first oxidative step of DTAA-based lipid synthesis with a secondary alcohol end group leads to the formation of a ketone chain end. Other than the carboxylic acid, the ketone moiety will not form the *N*,*N*-ditetradecylacetamide, which thus decreases the conversion and respective yield. Nevertheless, successful separation of ketone functional rPEG and the targeted lipid structure was feasible, yielding high-purity rPEG-DTAA. In addition, rPEG-DMG was synthesized in a four-step reaction (Fig. 1B) by (i) base-catalyzed tosylation of the hydroxy *ω*-end group. (ii) A dihydroxy glycerol *ω*-end group was introduced *via* functionalization with solketal and (iii) subsequent acidic acetal cleavage. Lastly, (iv) rPEG-dihydroxy glycerol was functionalized with myristoyl chloride in a Schotten–Baumann reaction, resulting in rPEG-DMG with high purity.

**Fig. 2:**
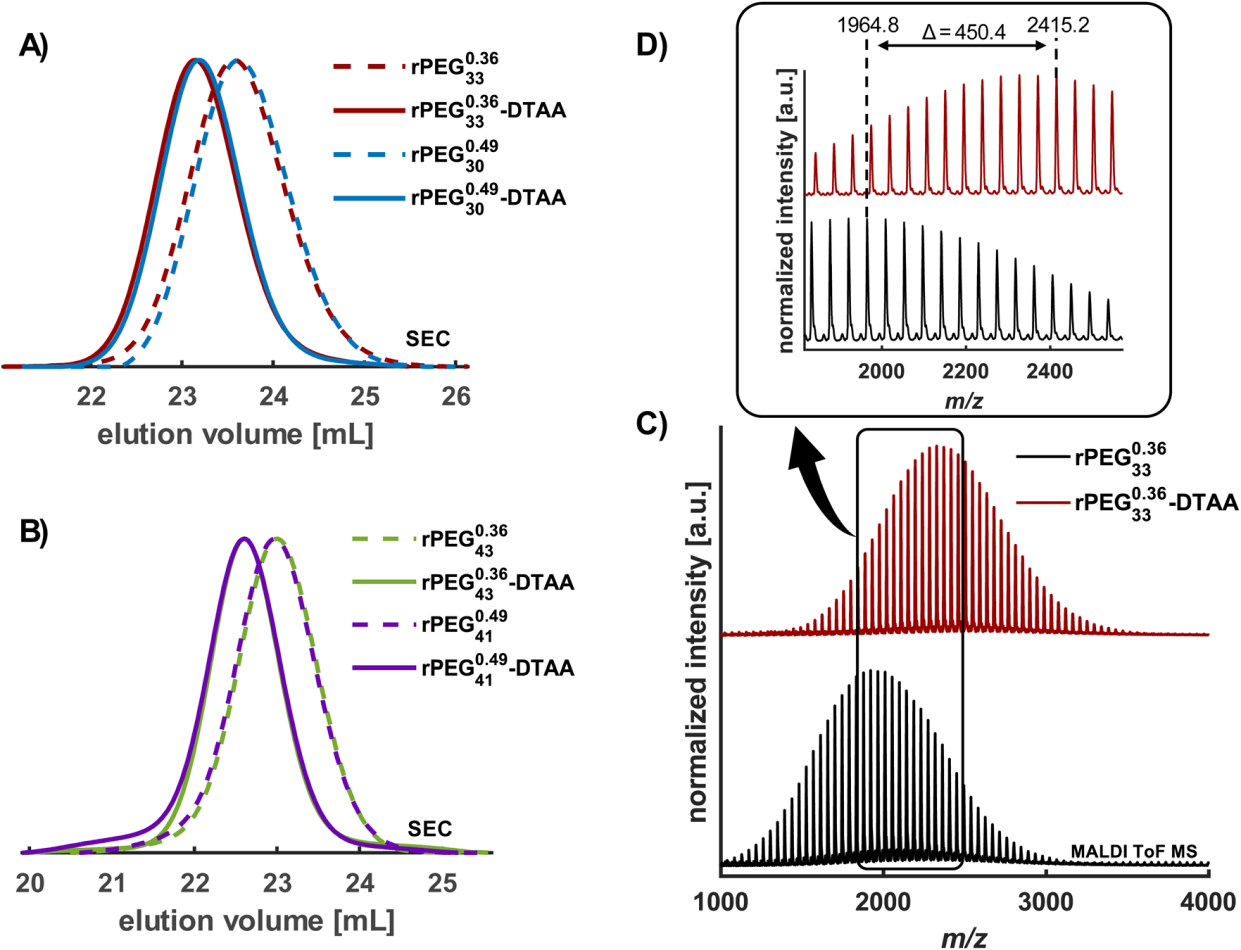
Characterization of rPEG-DTAA. (A) Stacked SEC traces of rPEGs and rPEG-DTAAs with constant *M*_n_; (B) stacked SEC traces of rPEGs and rPEG-DTAAs with constant DP; (C) exemplary stacked MALDI-ToF mass spectra of rPEG^0.36^_33_ and rPEG^0.36^_33_-DTAA; (D) zoom-in of stacked MALDI-ToF mass spectra of rPEG^0.36^_33_ and rPEG^0.36^_33_-DTAA.

All polymer lipid samples were purified *via* semi-preparative high performance liquid chromatography (HPLC), in accordance to the well-established purification method for pharma-grade PEG lipids. The successful synthesis of rPEG-DMG and rPEG-DTAA as well as the absence of precursors and side products were verified by size-exclusion chromatography (SEC), nuclear magnetic resonance (NMR) spectroscopy (Fig. S7–S10) and analytical reversed phase (RP) HPLC (Fig. S25 and S26). For the latter, purities of >99% for the rPEG lipids were determined. The increase in molar mass, coupled with the concurrent maintenance of a low dispersity, was confirmed through SEC (Fig. 2A, B, S17–S18) and matrix-assisted laser desorption/ionization time-of-flight mass spectroscopy (MALDI-ToF MS) (Fig. 2C, S11–S15). In addition, the MALDI-ToF MS further confirms the absence of rPEG precursors and high end group fidelity and purity of the rPEG lipids. Exemplary for rPEG^0.36^_33_-DTAA, the difference in *m*/*z* values between [rPEG + K]^+^ and [rPEG-DTAA + K]^+^ was found to be 450.4 g mol^−1^, which is close to the theoretical value of 450.8 g mol^−1^ (for *M*(linker + lipid)) (Fig. 2D).

### Recognition of rPEG lipids by APAs

**Fig. 3:**
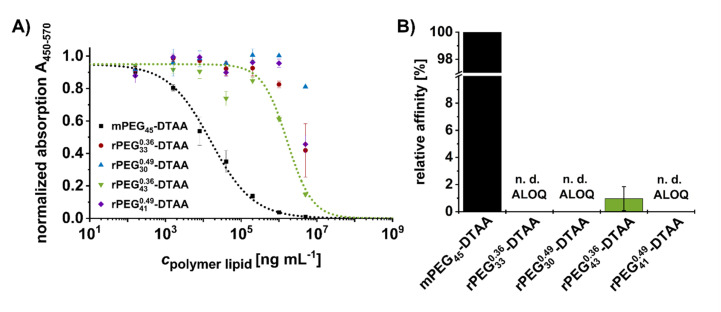
Interaction of mPEG and rPEG lipids and backbone-specific APA (6.3). (A) Competitive ELISA of mPEG- and rPEG-DTAA (160–5 × 10^6^ ng mL^−1^); (B) relative APA affinities normalized to the EC_50_ of mPEG-DTAA. The relative affinities of rPEG^0.36^_33_-DTAA, rPEG^0.49^_30_-DTAA, and rPEG^0.49^_41_-DTAA are not determined (n. d.) because their EC_50_ are above the highest concentration and can consequently be considered as above limit of quantification (ALOQ).

Following the successful synthesis of structurally varied rPEG lipids, the samples were examined for their interaction with APAs. A competitive enzyme-linked immunosorbent assay (ELISA) was performed using a backbone-specific 6.3 APA (murine IgG_1_). The competitive APA interactions of all synthesized rPEG-DTAAs were compared with those of mPEG-DTAA. Their EC_50_ (half maximal effective concentration) values were derived from a 4-parameter logistic regression. For further comparison, the relative APA affinities were normalized to the mPEG-DTAA reference. All rPEG-DTAAs demonstrated a drastic reduction in relative APA affinity of >99% (Fig. 3, Table S9). For rPEG^0.36^_33_-DTAA, rPEG^0.49^_30_-DTAA, and rPEG^0.49^_41_-DTAA, minimal APA recognition across the tested concentration was observed, and no sigmoidal regression could be applied. Therefore, the determination of an EC_50_ value was not possible, and the respective relative affinities were not determined (n.d.). Notably, rPEG lipids with 36 mol% GME were recognized at slightly lower concentrations than those with 49 mol% GME. This confirms that a higher degree of randomization suppresses APA binding more effectively. Similarly, all tested rPEG-DMGs demonstrated a drastically reduced relative APA affinity (Fig. S27, Table S10).

**Fig. 4:**
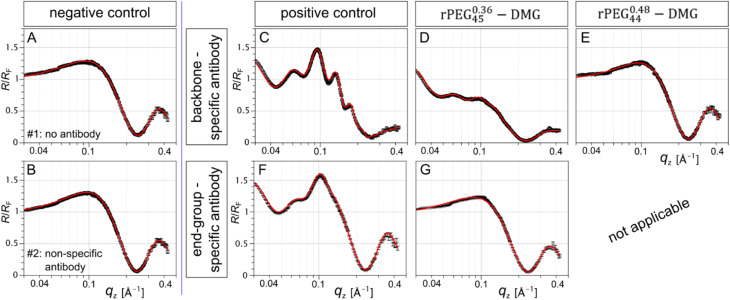
X-ray reflectivity curves (A)–(G) obtained with different PEG lipids and antibodies. A GME content of 36 mol% was sufficient to suppress the binding of end-group-specific antibodies, as the reflectivity curve for rPEG^0.36^_45_-DMG is identical to the negative control. Therefore, measurements of rPEG^0.48^_44_-DMG with a higher GME content of 48 mol% were not conducted with this antibody type (labeled “not applicable”).

To further confirm the absence of specific binding between rPEG lipids and APAs X-ray reflectometry (XRR) was utilized as an additional method under conditions that closely resemble the surfaces of lipid-based PEGylated drug delivery systems such as LNPs. Since LNPs have a diameter in the range of 100 nm (see below) they are much larger than the lipids, and their surface appears flat to lipids. Therefore, planar monolayers are suitable to mimic the surface of LNPs. XRR was exemplarily applied to monolayers of rPEG^0.36^_45_-DMG and rPEG^0.48^_44_-DMG. For this purpose, lipid monolayers containing 90 mol% 1,2-dipalmitoylphosphatidylcholine (DPPC) and 10 mol% rPEG-DMG were deposited onto the planar surfaces of aqueous solutions, as described in the Methods section. The utilized molar fraction is consistent with previously employed ratios for the investigation of APA/PEG interactions.^21,22^ The monolayers were laterally compressed to a surface pressure of 30 mN m^−1^, which coincides with a packing density as in a tensionless lipid layer under water.^34^ After compression, APAs were injected underneath the monolayer, as described in the Methods section, so that the final APA concentration was 0.01 mg ml^−1^. As positive control, mPEG_45_-DMG and two types of negative controls were used, (#1) an antibody-free aqueous solution and (#2) an aqueous solution of a non-specific IgG antibody, *i.e.*, an antibody of the same isotype as the APAs that does not selectively bind to PEG. XRR was then measured after at least 15 h incubation time. An overview of all reflectivity curves obtained with different PEG lipids and antibodies, including the positive and negative controls, is presented in Fig. 4. The shape of the reflectivity curves *R*/*R*_F_ (where *R*_F_ is the reflectivity of an idealized air/water interface) encodes the electron density profiles perpendicular to the air/water interface. The presence of the lipid monolayer at the air/water interface results in a characteristic electron density profile with a low-density region representing the alkyl chains and a higher-density region representing the phosphatidylcholine headgroups, followed by the constant density of water. The polyether chains are essentially invisible because their electron density is very similar to that of water. In contrast, the antibodies (proteins in general) have a significantly higher electron density than water, so that their binding or adsorption to the interface leads to additional modulations in the electron density profile and thus also in the reflectivity curves.

**Fig. 5:**
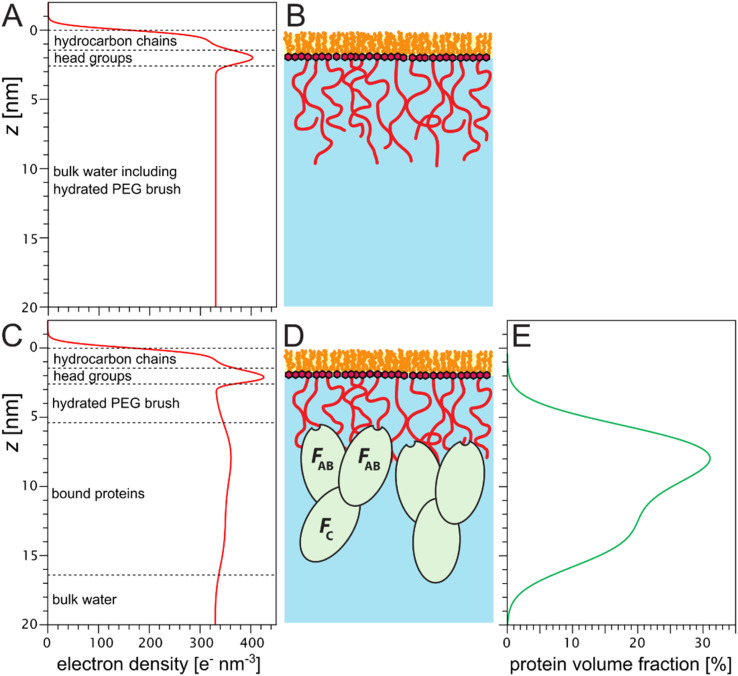
(A and C) Electron density profiles reconstructed from the X-ray reflectivity curves of lipid monolayers containing mPEG_45_-DMG in the absence of antibodies (A) and after incubation in the presence of end group-specific APAs (C); (B and D) schematic illustrations of the monolayer architecture and the corresponding antibody binding configuration; (E) protein volume fraction profile reconstructed from the electron density profile in panel (C).

The reflectivity curve of the monolayer containing mPEG_45_-DMG in the absence of antibodies (negative control #1, Fig. 4A) corresponds to the characteristic structure of a neat lipid monolayer. As the only significant feature, it exhibits one pronounced intensity minimum at around *q*_*z*_ = 0.25 Å^−1^. The reconstructed electron density profile with alkyl chain and headgroup layers is shown in Fig. 5A. The profile is consistent with a configuration schematically illustrated next to the plot on the right (Fig. 5B). Strikingly, the reflectivity curve of the monolayer containing mPEG_45_-DMG in the presence of non-specific antibodies (negative control #2, Fig. 4B) is virtually identical, demonstrating that non-specific antibodies do not adsorb non-specifically to the lipid layer.

**Fig. 6:**
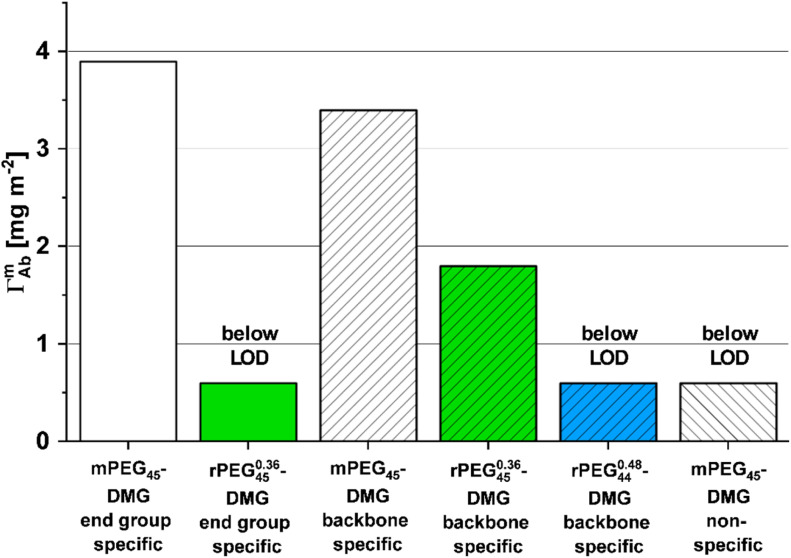
Bound antibody mass per unit area as obtained by integration of the protein volume fraction profiles for different mPEG and rPEG lipids and antibodies. The bound antibody mass of the end group-specific APA to rPEG^0.36^_45_-DMG, as well as the backbone specific APA to rPEG^0.48^_44_-DMG, and the non-specific AB to mPEG_45_-DMG, were below the limit of detection (LOD). The bound antibody mass of the end group-specific APA to rPEG^0.48^_44_-DMG has not been determined, as the binding to rPEG^0.36^_45_-DMG was already successfully suppressed, and the bound mass is below the LOD.

The reflectivity curves of the positive controls, a lipid monolayer containing conventional mPEG_45_-DMG on an aqueous solution of backbone-specific APA (Fig. 4C) or end group-specific APA (Fig. 4F), exhibit additional features that can be clearly attributed to antibody binding: reflectivity oscillations at lower *q*_*z*_-values (between 0.04 Å^−1^ and 0.2 Å^−1^) are indicative of the formation of thicker layers with elevated electron density. In simple terms, the *q*_*z*_-period of the oscillations reflects the layer thickness, while the oscillation amplitude increases with increasing electron density of the formed protein layers. The more antibody binding occurs, the more pronounced are these oscillations. A comparison of the reflectivity curves of the positive controls with end group-specific and backbone-specific APAs reveals differences, which results from the different distributions the antibodies assume in the different binding modes. This was shown in previous studies on brushes of mPEG using neutron reflectometry.^21^ For illustration and exemplification, Fig. 5C shows the reconstructed electron density profile for a lipid monolayer containing mPEG_45_-DMG exposed to end group-specific APAs. The bound APAs are seen as regions of elevated electron density. The electron density profile is consistent with a configuration schematically illustrated next to the plot on the right (Fig. 5D). The associated approximate protein volume fraction distribution, deduced as described in the Methods section, is shown in Fig. 5E. The width of the distribution (≈10–15 nm) can be understood as the overall extension of the protein layer and is consistent with the linear dimension of an IgG antibody. The maximum protein volume fraction of 25–30% is indicative of a relatively dense but not fully packed protein adsorption layer.^35^ Overall, the distribution is comparable in extension and maximum volume fraction to those previously reported for IgG antibodies bound to PEG brushes.^21,22^ Finally, the bound antibody mass per unit area, *Γ*^m^_Ab_, is obtained through integration of the volume fraction distribution, as described in the Methods section, and amounts to 3.9 mg m^−2^ in the illustrated example (Fig. 6).

Replacement of mPEG_45_-DMG with rPEG^0.36^_45_-DMG in the monolayer dramatically changes the reflectivity curve obtained after incubation of end group-specific APAs (Fig. 4G). In fact, the reflectivity curve is indistinguishable from the curve obtained for the two negative controls, demonstrating that antibody binding remains below the detection limit, which we conservatively estimate as <0.6 mg m^−2^ (Fig. 6). This result confirms that the chosen polymer architecture and GME content disturbs the binding of the end group-specific antibody. From a prior study, it has been established that interaction of end group-specific APA and PEG necessitates not only the presence of the methoxy group but also three additional consecutive ethylene oxide units.^32^ Additionally, while end group-specific antibodies are capable of binding to the polymer backbone, this interaction requires significantly increased concentrations.^20^ A moderate GME content of 36 mol% is sufficient to suppress binding of end group-specific APAs to surfaces that mimic those of LNPs or other drug delivery nanocarriers. It was therefore unnecessary to further increase the substitution ratio for experiments with end group-specific APAs and the binding of the end group-specific APA to rPEG^0.48^_44_-DMG was not further investigated. The reflectivity curve after incubation with backbone-specific APAs changes drastically, too, if mPEG_45_-DMG is replaced with rPEG^0.36^_45_-DMG. The oscillations at low *q*_*z*_ do, however, not entirely disappear (Fig. 4D), which demonstrates that some binding of backbone binding APAs remains at a GME content of 36 mol%. By increasing the GME substitution ratio to 48 mol% (*i.e.*, by replacing mPEG_45_-DMG in the monolayer with rPEG^0.48^_44_-DMG), the oscillations disappear completely (Fig. 4E), indicating undetectably low antibody binding. Fig. 6 summarizes the bound antibody mass per unit area, *Γ*^m^_Ab_, for all samples investigated. The recognition of rPEG^0.36^_45_-DMG by backbone-specific APAs but not by end-group specific APAs may be rationalized by the higher probability of finding consecutive EO repeating units in the backbone as compared to the chain end.

### Lipid nanoparticle formulation and *in vitro* transfection of LNPs containing rPEG lipids

It must be ensured that rPEG lipids can be formulated into LNPs with the same properties and performance as LNPs based on PEG lipids to benefit from the favorable antigenic properties of rPEG lipids in RNA delivery. Relevant physico-chemical properties for lipid nanoparticles are the size, the polydispersity index (PDI), the zeta potential, and the RNA encapsulation efficiency, whereas the performance of the particles can be evaluated by means of the transfection efficiency.

**Fig. 7:**
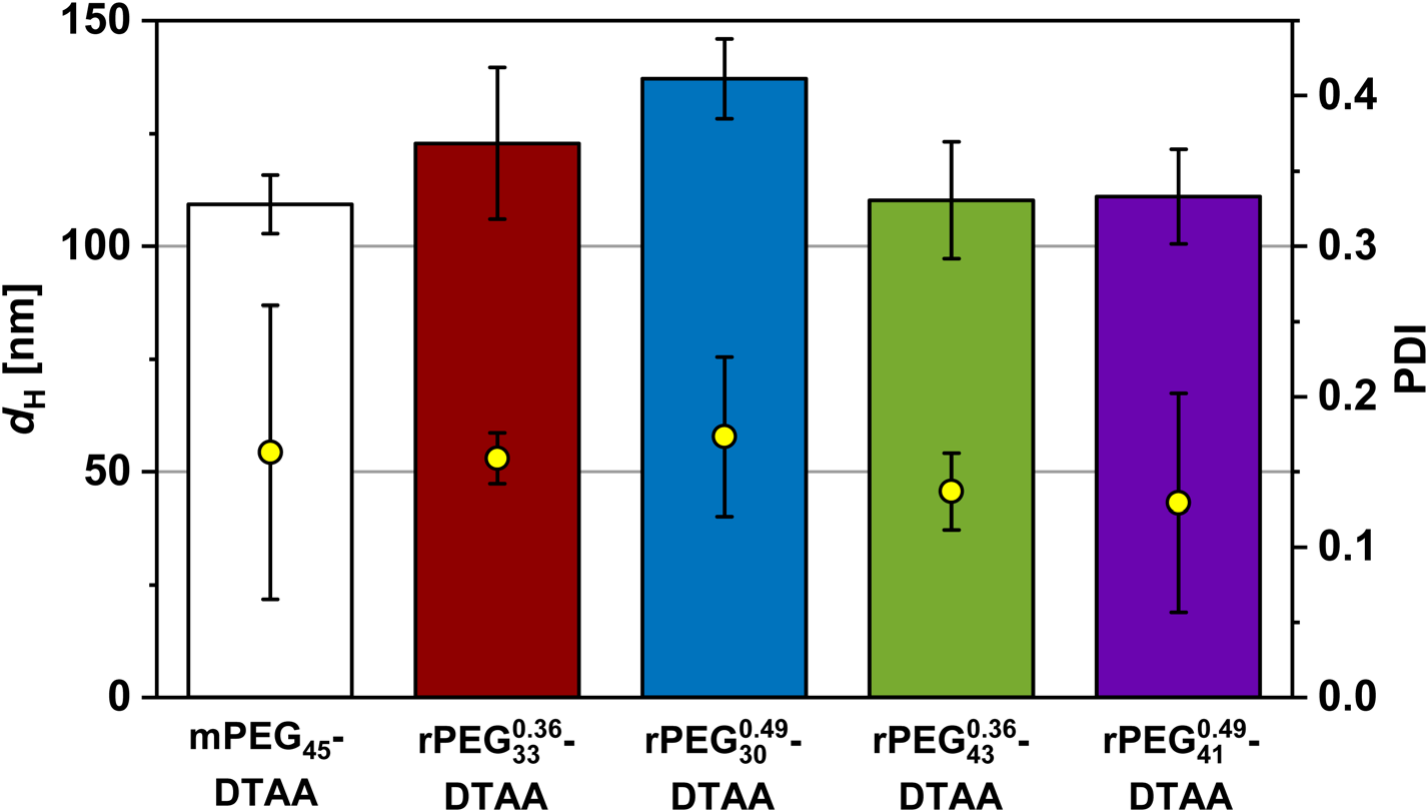
Physico-chemical characterization of LNP formulations determined *via* DLS. Hydrodynamic diameters (bars) and PDIs (yellow circles) of mPEG_45_-DTAA and rPEG-DTAAs.

LNP formulations containing rPEG-DTAA as a stealth lipid and firefly luciferase mRNA (FLuc mRNA) as a cargo were prepared and compared to their respective PEG analogues. Characterization of the obtained rPEG-DTAA LNPs revealed a similar mean diameter of the PEG and rPEG LNPs between 109 and 137 nm and PDIs of 0.13–0.18 (Fig. 7). In addition, the zeta potentials of the LNPs containing rPEG-DTAA and mPEG_45_-DTAA were found to be similar both before (43.0 to 52.6 mV) and after dialysis (−14.3 to −8.8 mV) (Table S3).

**Fig. 8:**
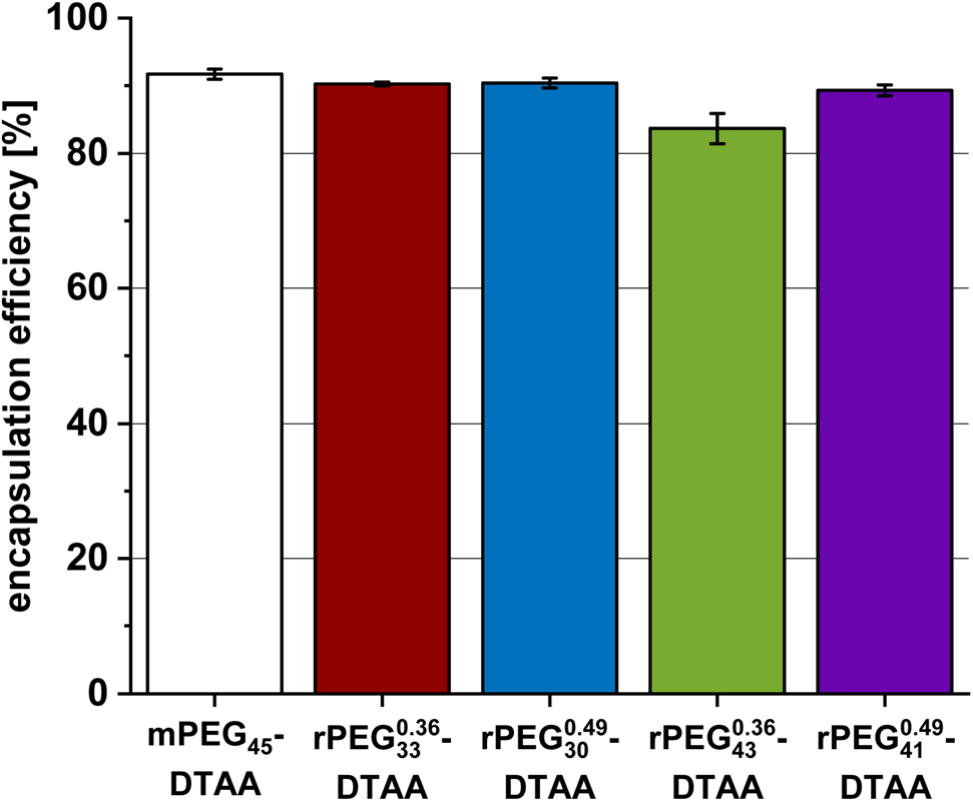
RiboGreen assay of LNPs containing mPEG_45_-DTAA and rPEG-DTAA.

It was observed that LNPs containing rPEG-DTAA with a DP similar to that of mPEG_45_-DTAA had mean diameters and zeta potentials closely resembling the mPEG_45_-DTAA formulations. A table containing the particle size, PDI, and zeta potential of the obtained LNPs can be found in the SI (Table S3). The results suggest that the main contribution to the size of the nanoparticles derives from the hydration of the backbone, while it is independent of the amount of methoxymethylene side groups within the investigated compositions. This is in accordance with the results observed for the hydrodynamic radii of rPEGs with constant DP and varying GME content.^32^ The mRNA encapsulation efficiency was assessed using agarose gel electrophoresis (AGE) and RiboGreen assay. Both methods confirmed comparable mRNA encapsulation efficiency for all rPEG-DTAAs and mPEG_45_-DTAA (Fig. 8), ranging from 83% to 91%. In a similar manner, mPEG_45_-DMG and rPEG-DMG-based LNP formulations also showed similar RNA encapsulation efficiencies, too (Table S8).

**Fig. 9:**
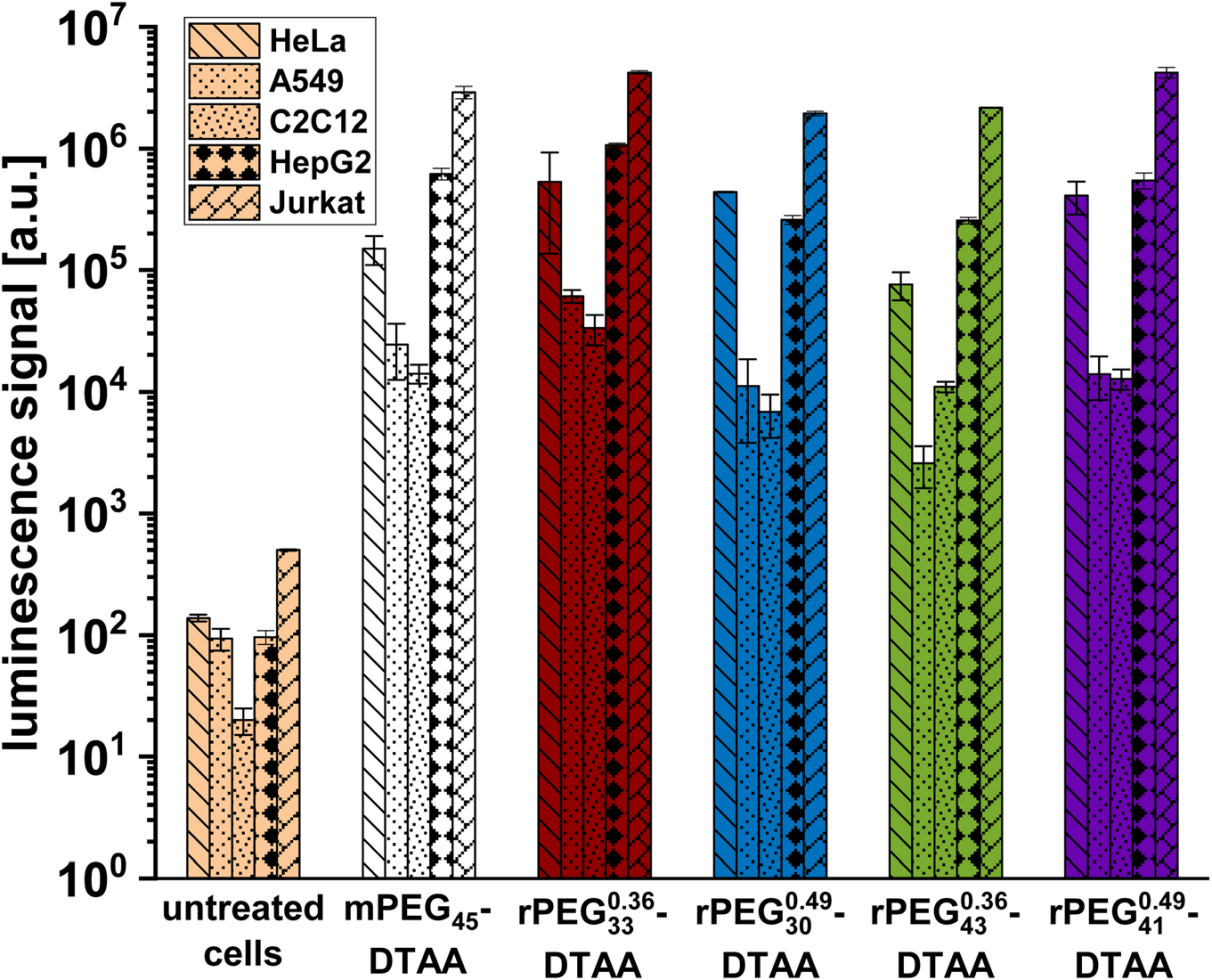
Luciferase assay of LNPs containing mPEG_45_-DTAA and rPEG-DTAA. The diagram shows the luminescence for 5 cell lines, as specified in the inset.

To compare the performance of all LNPs, the transfection efficiency was evaluated across various human cell lines including HeLa, Jurkat, C2C12, HepG2, and A549 using a luciferase assay (FLuc mRNA). Overall, rPEG lipid-based formulations achieved transfection efficiencies comparable to those of established PEG lipids (Fig. 9), as demonstrated by the similar levels of luciferase expression detected. It can thus be concluded that rPEG lipid formulations are taken up by cells and effectively deliver mRNA into a variety of cells in full analogy to clinically established mPEG lipid formulations.

### Cytotoxicity of lipids and LNPs based on rPEG-lipids

**Fig. 10:**
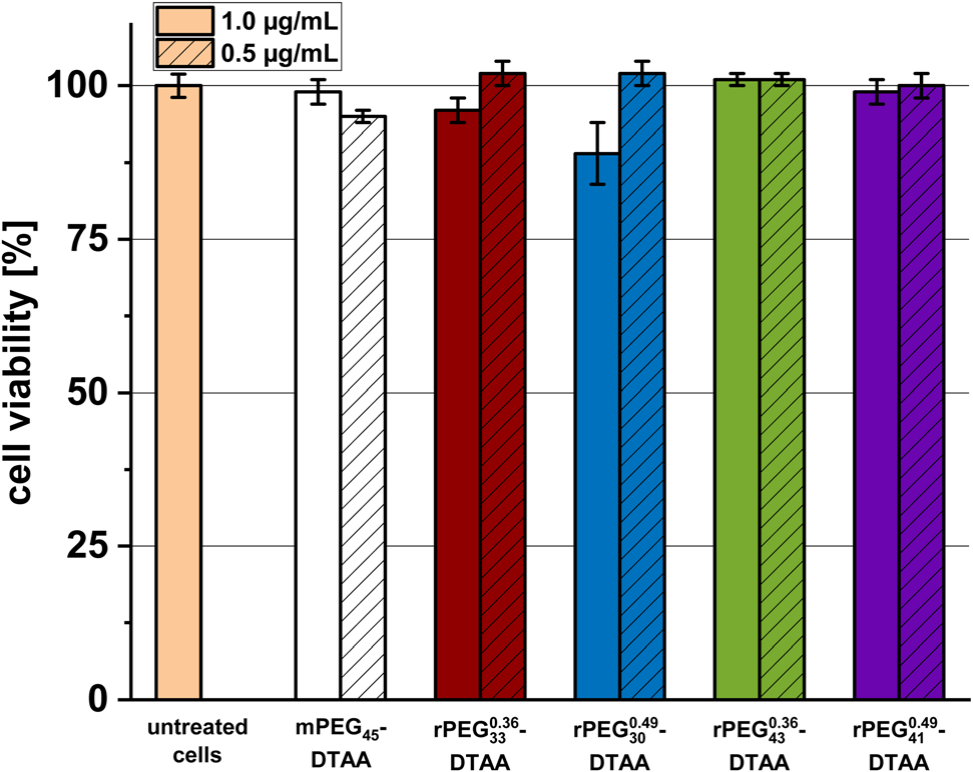
Comparison of cell viability based on MTS assay of LNPs containing mPEG_45_-DTAA or rPEG-DTAA.

Cell viability of rPEG lipids and rPEG LNPs was investigated *via* 3-(4,5-dimethylthiazol-2-yl)-5-(3-carboxymethoxyphenyl)-2-(4-sulfophenyl)-2*H*-tetrazolium (MTS) assays (Tables S4–S7). The cytotoxicity assays showed that cells treated with rPEG lipids or LNPs maintained a higher or similar viability as cells treated with the PEG-based lipid or formulation, respectively (Fig. 10, S23 and S24). In detail, the viability of treated cells remained above 89% in both formulations for concentrations between 0.5–1.0 µg mL^−1^ indicating that all rPEG formulations enable high delivery efficiency without compromising cell health and cell metabolism.

Optical microscopy analysis (Fig. S22) was conducted to visually assess cell viability in both untreated and treated samples indicating that the treatment did not induce visible signs of cytotoxic stress, such as cell rounding, membrane blebbing, or detachment. The microscopy results align with the findings from standard cytotoxicity assay, also confirming that rPEG formulation does not compromise cell health under the applied assay conditions.

## Conclusion

To address the need for PEG alternatives in lipid-based drug delivery, a PEG isomerization (rPEG) approach for the synthesis of novel polymer-lipids has been implemented. As a proof of concept, we demonstrate that rPEG formulations exhibit physico-chemical properties and performance comparable to those of established PEG-based LNP formulations. ELISA and XRR measurements of rPEG lipids with 36 mol% and 49 mol% GME content, which can be referred to as moderate and high degree of randomization, respectively, further confirm their significantly reduced antigenicity at pharmaceutically relevant concentrations against different types of anti-PEG antibodies. rPEG lipids not only address growing concerns regarding PEG immunogenicity but also maintain the critical attributes essential for effective drug delivery. The combined results demonstrate the suitability of rPEG-lipid containing LNPs for RNA delivery and constitute a relevant advancement in the design of safer and more versatile drug delivery nanocarrier systems. In future *in vivo* studies we aim at an investigation of the blood circulation times of rPEG lipid-containing LNPs after repeated dosing. We will also study whether the random incorporation of GME can prevent the formation of anti-rPEG antibodies *in vivo* to establish rPEG as a promising PEG alternative.

## Methods

Experimental details can be found in the SI.

## Author contributions

P. D. and C. B. contributed equally. The manuscript was written through the contributions of all authors. All authors have given approval to the final version of the manuscript.

## Conflicts of interest

P. Dreier, R. Matthes, and H. Frey are listed as co-inventor of two patents regarding rPEG and rPEG-lipids (WO2024/105084 and WO2024/105068). J. Scheiger is an employee of Evonik Operations GmbH, which is the applicant of the patent WO2024/105068. Evonik Operations GmbH partners with the University of Mainz to commercialize rPEG lipids.

## Supplementary Material

SC-017-D6SC00169F-s001

## Data Availability

The data supporting this article have been included as part of the extended supplementary information (SI). Supplementary information: reagents and equipment, Methods, NMR spectroscopy, size exclusion chromatography, MALDI-ToF MS, HPLC, ELISA, XRR, LNP formulation, DLS, MTS Assay, Luciferase Assay, Ribogreen Assay, Synthesis of GME, MMEPOH, the reported copolymers, and the lipids, characterization of the reported compounds. See DOI: https://doi.org/10.1039/d6sc00169f.
