# Supplementary material for: Lipids and lipid nanoparticles functionalized with randomized poly(ethylene glycol) (rPEG) for mRNA delivery
Source: Chem Sci. 2026 May 29;17(28):14064–73. doi: 10.1039/d6sc00169f (PMC13266465; doi:10.1039/d6sc00169f)
Supplement: SC-017-D6SC00169F-s001 [file SC-017-D6SC00169F-s001.pdf]

Supplementary Information (SI) for Chemical Science.
This journal is © The Royal Society of Chemistry 2026

-Supporting Information-

Lipids and Lipid Nanoparticles Functionalized with Randomized Poly(Ethylene Glycol) (rPEG) for mRNA Delivery

Philip Dreier[1, 2, ‡], Caroline Bockhard[1, ‡], David Seibel[4], Olaf Soltwedel[4], Rebecca Matthes[1], Gregor M. Linden[1], Julian Schmidt[1] Dominik Göbel[3], Thomas Endres[2], Johannes Scheiger[2], Regine von Klitzing[4], Emanuel Schneck[4] and Holger Frey[1, *]

[1]Department of Chemistry, Johannes Gutenberg-University Mainz, Duesbergweg 10-14, 55128 Mainz, Germany

[2]Evonik Operations GmbH, Kirschenallee, 64293 Darmstadt, Germany

[3]Evonik Operations GmbH, Rodenbacher Chaussee 4, 63457 Hanau, Germany

[4]Institute for Condensed Matter Physics, Technische Universität Darmstadt, Hochschulstrasse 8, 64289 Darmstadt, Germany

[‡]PD and CB contributed equally to this work.

1. Materials and Instrumentation

1.1. Reagents and equipment

All chemicals and solvents were purchased from Acros Organics, Roth, TCI, Sigma-Aldrich, Fisher Scientific, Fluka, Carl Roth GmbH, Merck KGaA, and Orgentis Chemicals (Gatersleben, Germany), unless otherwise noted. Deuterated solvents were received from Deutero GmbH. Ethylene oxide was acquired from Air Liquide. THF was flashed over basic aluminum oxide before usage. Glycidyl methyl ether was dried over CaH_2 and cryo-transferred before polymerizations. CleanCap® FLuc mRNA was obtained from TriLink BioTechnologies (San Diego, CA, USA). D-Lin-MC3-DMA were obtained from MedChemExpress (Monmouth Junction, NJ, USA), cholesterol and PEG2k-DMG were purchased at Merck KGaA, DSPC was obtained from NOF (White Plains, NY, USA). All cell lines were supplied by German Collection of Microorganisms and Cell Cultures GmbH (DSMZ, Braunschweig, Germany). $mPEG_{45}$-DMG and $mPEG_{45}$-DTAA were provided by Evonik. Both end group specific (Anti-methoxy-PEG Monoclonal, Clone 5D6-3) and backbone specific (Anti-PEG Monoclonal, Clone 9B5-6-25-7) anti-PEG antibodies used for the X-ray reflectometry were received from Life Diagnostics (West Chester PA, USA) and the non-specific antibody (Mouse IgG1 kappa Isotype Control (P3.6.2.8.1)) from eBioscience™, Thermo Fisher Scientific (Carlsbad CA, USA). The primary backbone specific anti-PEG antibody 6.3 used in the ELISA measurements was purchased from IBMS Academia Sinica (Taipeh, Taiwan) and the detection antibody

(horseradish peroxidase conjugated AffiniPure donkey anti-mouse IgG (H+L) 1:10000) was supplied by Immuno Jackson Research (Ely, UK)

1.2. NMR spectroscopy

^{1}H and ^{13}C NMR spectra were recorded on a Bruker Avance III HD 400 spectrometer with 400 and 100 MHz, respectively, and referenced internally to residual proton signals of the deuterated solvent. All spectra were acquired at 23 °C. Spectra were processed and analyzed utilizing the MestReNova 14.3.3-33362 software.

1.3. Size exclusion chromatography (SEC)

Measurements were conducted using an Agilent 1100 series HPLC system, which included a degasser, isocratic pump (G1310A), autosampler (G1313A), column oven (G1316A), and detectors for refractive index (RI) (G1310A) and variable wavelength (VWD) (G1314A). Separations were carried out employing a four-column set-up (MZ-Analysentechnik GmbH) connected sequentially:

i) HEMA-40 guard column (40 Å pore size, 10 µm particle size, 50x8.0 mm)
ii) HEMA-40 analytical column (40 Å pore size, 10 µm particle size, 300x8.0 mm)
iii) HEMA-100 analytical column (100 Å pore size, 10 µm particle size, 300x8.0 mm)
iv) HEMA-300 analytical column (300 Å pore size, 10 µm particle size, 300x8.0 mm)

The eluent consisted of DMF (Fisher Chemical) with 1 mg mL^{-1} anhydrous LiBr (Acros Organics), delivered at a flow rate of 1 mL min^{-1}. Both, the column oven and RI detector cell were maintained at 50 °C. Calibration was performed using well-defined poly(ethylene glycol)s from PSS (PSS Standards Kit) with molecular weight values (M_p) ranging from 106 to 42700 Da. Samples were dissolved in DMF (with 1 mg mL^{-1} anhydrous LiBr) at a concentration of 1 mg mL^{-1} with the addition of 1 drop of toluene. Injection of 100 µL of the stock solutions was carried out via the autosampler, with a measurement duration of 45 min. Elution times were referenced using toluene as an internal standard. RI traces were analyzed using the PSS WinGPC Unichrom V8.31 software.

1.1. MALDI-ToF mass spectrometry (MS)

MALDI-TOF MS measurements were carried out at a Bruker autoflex maX MALDI-TOF/TOF using a smartbeam-II solid state laser with a wavelength of 337 nm. Spectra were recorded using the software Bruker flexControl 3.4 and analyzed using Bruker flexAnalysis 3.4 and Bruker polytools 1.31. The potassium salt of trifluoroacetic acid (KTFA) and trans-2-[3-(4-*tert*-butylphenyl)-2-methyl-2-propenylidene]malononitrile (DCTB) were utilized as ionization salt and matrix, respectively. For sample preparation the polymers were dissolved in chloroform at 10 mg mL^{-1}. 20 µL of this solution were combined with 20 µL of a 10 mg mL^{-1} solution of the matrix in chloroform. 5 µL of a 0.1 M solution of the salt in methanol were added and 1 µL of the resulting mixture was spotted onto a MTP 384 ground steel target plate. The solvents were allowed to evaporate completely before the measurement. All measurements were performed in linear mode.

1.2. Analytical high-performance liquid chromatography (HPLC)

rPEG-DMG: The HPLC system consisted of an Agilent Technologies 1260 Infinity system with a 1260 Quat pump with solvent degasser, a 1260 ALS autosampler, a 1260 column thermostat, a Thermo Fisher Corona Veo RS Charged Aerosol Detector (CAD). The CAD was operated with an evaporator temperature of 30 °C, a power function of 1.3, a data collection rate of 2 Hz and a filter constant of 3.6 s. The column oven temperature was set to 40 °C. A flow rate of 1 mL min^{-1} was applied. For analysis, a Poroshell 120 (Phenylhexyl , 2.7 µm particle size, 150 × 4.6 mm (L × ID) column from Agilent was used. The mobile phase A consisted of 75 % (v/v) water (LiChrosolv® Merck Chemicals, LC-MS grade) with 0.1% TFA (v/v) (HiPerSolv Chromatogram LC/MS VWR) and 25% (v/v) acetonitrile (LiChrosolv® Merck Chemicals, LC-MS grade). The mobile phase B consisted of 70% (v/v) acetonitrile (LiChrosolv® Merck Chemicals, LC-MS grade) and 30% (v/v) methanol (LiChrosolv® Merck Chemicals, LC-MS grade).

Samples were dissolved in H_2O/THF 2:1 (v/v) at a concentration of 5 mg mL^{-1}. 5 µL of the stock solution was injected and analyzed by RP-HPLC with a MeOH/H_2O/ACN/MeOH gradient:

i) 0 min: 75% A; 25% B
ii) 0-20 min: linear gradient to 100% B
iii) 20-25 min: isocratic at 100% B

Subsequently, the starting gradient was restored within 0.1 min and a 5 min reconditioning time at 75% A; 25% B was allowed before further analysis.

rPEG-DTAA: The HPLC system consisted of an Agilent Technologies 1260 Infinity system with a 1260 Quat pump with solvent degasser, a 1260 ALS autosampler, a 1260 column thermostat, a Thermo Fisher Corona Veo RS Charged Aerosol Detector (CAD). The CAD was operated with an evaporator temperature of 30 °C, a power function of 1.3, a data collection rate of 5 Hz and a filter constant of 2.0 s. The column oven temperature was set to 40 °C. A flow rate of 1 mL min^{-1} was applied. For analysis, a ZORBAX SB-C8 (1.8 µm particle size, 75 × 4.6 mm (L × ID) column from Agilent was used. The mobile phase A consisted of water (LiChrosolv® Merck Chemicals, LC-MS grade) with 0.1% TFA (v/v) (HiPerSolv Chromatogram LC/MS VWR). The mobile phase B consisted of 95% (v/v) tetrahydrofuran (LiChrosolv® Merck Chemicals, LC-MS grade) and 5% (v/v) water (LiChrosolv® Merck Chemicals, LC-MS grade) with 0.1% TFA (v/v) (HiPerSolv Chromatogram LC/MS VWR). Samples were dissolved in H_2O/THF 4:1 (v/v) at a concentration of 5 mg mL^{-1}. 20 µL of the stock solution was injected and analyzed by RP-HPLC with a H_2O/THF/H_2O gradient:

i) 0-0.5 min: isocratic at 90% A; 10% B
ii) 0.5-4.5 min: linear gradient to 30% A; 70% B
iii) 4.5-12 min: linear gradient to 5% A; 95% B
iv) 12-14 min: isocractic at 5% A; 95% B

Subsequently, the starting gradient was restored within 0.5 min and a 5 min reconditioning time at 90% A; 10% B was allowed before further analysis.

1.3. Enzyme-linked immunosorbent assay (ELISA)

We established a customized ELISA protocol for rPEG samples[1] adapted from the protocols reported by Roffler et al.[2] In detail, Maxisorp 96-well plates (Thermo Fisher Scientific) were coated with 0.5 µg per well mPEG114-amine (Biopharma PEG) in $NaHCO_3$/Na_2CO_3 buffer (0.1 M, pH 9.5) overnight at 4 °C. The coating solution was discarded, and the plate was washed three times with 300 µL PBS per well. Blocking was performed using 5% (w/v) skim milk powder in PBS for 2 hours at 21 °C, followed by one washing step with 300 µL PBS. 50 µL of the competitive lipids (160 – 5 x 10^6 ng mL^{-1} in PBS) were added in duplicate, as well as 50 µL of PBS as a negative control (12 wells) and incubated for 30 min at 21 °C with shaking at 300 rpm. 6.3 (IBMS Academia Sinica, Taiwan) was diluted with 4% (w/v) skim milk powder in PBS at a final concentration of 50 ng mL^{-1}. 50 µL of the primary antibody mixture was added to the plate and incubated (1 h, 21 °C, 300 rpm). Note: The analyzed concentrations of the competitive samples and the primary antibody are reduced by a dilution factor of 2. Unbound antibodies, antibodies bound to the competitive analyte, as well as unbound analytes, were removed by washing with 2 x 300 µL 0.1% (w/v) CHAPS/PBS (3-((3-Cholamidopropyl)dimethylammonio)-1-propanesulfonate) and 1 x PBS. Detection was performed using 50 µL horseradish peroxidase conjugated AffiniPure donkey anti-mouse IgG (H+L) (1:10000, Immuno Jackson Research) for 1 hour at 21 °C with 300 rpm shaking, followed by washing with 4 x 300 µL 0.1% (w/v) CHAPS/PBS and 2 x 300 µL PBS. Subsequently, 100 µL of 1-Step TMB ELISA Substrate Solution (Thermo Fisher Scientific) was incubated for 20 minutes at 21 °C with shaking at 300 rpm. The reaction was stopped with the addition of 100 µL H_2SO_4 (2 M). Absorbance was measured using a FLUOstar Omega multi-mode reader (BMG Labtech) at 450 nm (OD_{450}), with reference reading at 570 nm (OD_{570}) subtracted for background correction. The corrected data were normalized to the mean of 12 PBS controls. Half-maximal effective concentrations (EC_{50}) were derived using a four-parameter logistic (4PL) sigmoidal regression model

using Origin2024 Pro. Subsequently, the relative affinities of the samples were determined by normalization to the reference sample (mPEG-DTAA or mPEG-DMG) with error propagation.

1.4. X-Ray reflectometry from PEG-DMG-containing lipid monolayers exposed to antibody solution

1.4.1 Monolayer preparation and injection of antibody solutions

For the preparation of the Langmuir monolayer, stock solutions of DPPC, $mPEG_{45}$-DMG, $rPEG_{45}^{0.36}$-DMG, and $rPEG_{44}^{0.48}$-DMG were first prepared in chloroform at concentrations of 1 mg ml^{-1}. These stock solutions were mixed to produce a solution containing 10 mol% of the particular PEG lipid and 90 mol% of 1,2-Dipalmitoylphosphatidylcholine (DPPC, Merck, Darmstadt, Germany). To obtain the Langmuir monolayer, 15 ± 2 µL of the mixed solution was spread on 23 ml of the aqueous buffer in a Teflon trough with a surface of 40 cm^2. The resulting surface pressure was 30 ± 2 mN m^{-1}, measured with the Wilhelmy plate method using a paper sensor. For the measurements with antibodies, the different antibody buffer solutions (either end group specific, backbone specific or non-specific) were first mixed with the HEPES buffer solution, always yielding a concentration of 0.23 mg mL^{-1}. A liquid volume of 1 mL of the subphase was then extracted from the trough through a hole in one of the side walls. This volume was then replaced by injecting 1 mL of the mixed antibody solution through the same hole. In order to facilitate mixing of the aqueous medium during injection, the needle was gently moved from one side to the other while pulling it out of the hole, so that the injection was evenly distributed over the liquid volume. The resulting antibody concentration in the subphase was 0.01 mg mL^{-1}. XRR measurements were carried out after an incubation time of at least 15 hours, which can be considered sufficient for the complete diffusive homogenization of the antibody concentration in the solution and to saturate the binding of antibodies to the PEG-decorated surface. In fact, in earlier reflectometry studies using liquid cells, an equilibration time of 6 hours was found to be sufficient.[3]

1.4.2 X-ray reflectometry (XRR) experiments

The measurements were carried out with a D8 diffractometer (Bruker AXS, Karlsruhe, Germany) in which a monochromatic beam with a photon wavelength of 1.541 Å was collimated to a horizontal slit with a height of 0.1 mm. The reflectivity was corrected for the beam footprint and angular misalignment and was normalized on the incident beam intensity. The initially unknown parameters for the footprint correction were reconstructed from the requirement that the known electron density value of the hydrocarbon chain layer was correctly reproduced. For each measurement with the different antibody solutions, the XRR measurement was first performed with the lipid monolayer on the buffer without antibodies. The antibodies were then injected, and the next measurement was started after an incubation time of 15 hours or more, see main text.

1.4.3 XRR analysis

To determine the electron density profiles, the experimentally determined reflectivity curves $R(q_z)$ were compared with the theoretical reflectivity curves calculated on the basis of a slab model for the interfacial electron density profiles $\rho(z)$. This was done as explained in reference 4, from which the following text is partially reproduced.[4] The lipid monolayer film was described with two homogeneous slabs or boxes of adjustable thickness and electron density, which represent different portions of the lipid monolayers, namely headgroups and hydrocarbon tails. The adjacent semi-infinite bulk media air and water were modeled with constant electron densities. The interfaces between slabs were subject to interfacial roughness to an adjustable extent encoded in the roughness parameters. After antibody binding, the electron density profile model had to be extended by three additional slabs with adjustable parameters, one slab representing the antibody-free region of the PEG "brush" and two slabs to represent the layers of bound antibodies in the brush periphery. Mathematically, the electron density profiles are given as

$$\rho(z) = \rho_1 + \sum_{j=2}^{N+2} \frac{1}{2}(\rho_j - \rho_{j-1}) \left(1 + \mathrm{erf}\left(\frac{z - z_{j-1}}{\sqrt{2}\sigma_{j-1,j}} \right) \right) \qquad \textbf{(S1)}$$

, where N is the number of slabs, $\rho_1 = 0$ is the electron density of air, $\rho_{N+2} = \rho_W = 330$ nm^{-3} is the known electron density of water, ρ_2 to ρ_{N+1} are the electron densities of the slabs, $\sigma_{1,2}$ to $\sigma_{N+1,N+2}$ the roughness parameters of the interfaces between them, and z_1 to z_{N+1} are the positions of the slab boundaries, as determined by the slab thicknesses.

The electron density profiles were then discretized into hundreds of thin ideal sub-slabs of constant electron density. The corresponding q_z-dependent reflectivities, $R(q_z)$, were then calculated from the Fresnel reflection coefficients at each slab-slab interface using the iterative recipe by Parratt.[5] Starting from a set of initial parameters coming from an "educated guess", all model parameters are varied so that the theoretical reflectivity curve best matches the measured reflectivity curves in terms of the minimal χ^2-deviation.

1.4.4 Reconstruction of protein volume fractions

The electron density profile can also be represented in terms of the volume fraction profiles of all chemical components. At the periphery of the PEG brush, there are only three components to consider, namely water, PEG, and antibodies. Since PEG is a minority fraction that also has essentially the same electron density as water, only water and the antibodies have to be considered, which have the known electron densities ρ_W (see above) and $\rho_{Ab} = 430$ nm^{-3}.[6] With that, the electron density profile is locally given as

$$\rho(z) = \rho_{\text{Ab}}\Phi_{\text{Ab}}(z) + \rho_{\text{W}}\Phi_{\text{W}}(z) \quad \textbf{(S2)}$$

, where $\Phi_{Ab}(z)$ and $\Phi_W(z) = 1 - \Phi_{Ab}(z)$ are the volume fraction profiles of antibodies and water, respectively. This expression can be solved for $\Phi_{Ab}(z)$:

$$\Phi_{\text{Ab}}(z) = \frac{\rho(z) - \rho_{\text{W}}}{\rho_{\text{Ab}} - \rho_{\text{W}}} \quad \textbf{(S3)}$$

1.4.5 Calculation of the bound antibody mass

The integral over the volume fraction of bound antibodies yields the equivalent thickness.[3]

$$D_{\text{Ab}} = \int \Phi_{\text{Ab}}(z)\text{d}z \quad \textbf{(S4)}$$

With the mass of an IgG1k antibody of $m_{Ab} = 150$ kDa and its volume $v_{Ab} \sim 180$ nm^3 ,[3] this results in the total adsorbed antibody mass per unit area Γ_{Ab} with

$$\Gamma_{\text{Ab}} = \frac{m_{\text{Ab}}D_{\text{Ab}}}{v_{\text{Ab}}} \quad \textbf{(S5)}$$

1.5. LNP formulation

An aqueous phase containing 0.133 g L^{-1} FLuc mRNA and 11 mM acetic acid was mixed with an ethanolic phase containing 9.43 mM total lipid (50 mol% DLin-MC3-DMA, 38.5 mol% cholesterol, 10 mol% DSPC, 1.5 mol% PEG lipid or rPEG lipid at a 3:1 volume ratio. The crude LNP colloidal dispersion

was dialyzed against phosphate-buffered saline (PBS) for 3 h (3x buffer exchange). Purified LNPs were stored at 4 °C until further use.

1.6. Dynamic light scattering (DLS)

Measurements were conducted with a Zetasizer NanoZS from Malvern Instruments GmbH (Herrenberg, Germany). A DTS 1070 clear disposable folded capillary cell from Malvern Panalytical GmbH (Kassel, Germany) was used. For particle size measurement, samples were diluted 1:10 in RNAse free water corresponding to an RNA concentration of 5 ng μL^{-1}. For zeta potential (z-pot.) measurement, colloidal LNP dispersions were diluted 1:30 in RNAse free water corresponding to an RNA concentration of 1.67 ng μL^{-1}. Z-ave, the width of the fitted Gaussian distribution, which is displayed as the polydispersity index (PDI), as well as the average z-pot. values were calculated from data of at least 10 runs.

1.7. MTS assay

MTS (3-(4,5-Dimethylthiazol-2-yl)-5-(3-carboxymethoxyphenyl)-2-(4-sulfophenyl)-2H-tetrazolium) assay was conducted with either HeLa or HepG2 cells. One day before transfection, 10,000 cells per well were seeded into a 96 well plate in a volume of 100 µL of the respective medium (containing 10% FBS) and cultured for 24 h at 37 °C and 5% CO_2. On day 2, old medium was removed and 90 µL of fresh medium was added to the cells. For polymer testing, compounds were dissolved in sterile water to meet the targeted concentrations. Samples were added within a volume of 10 µL. For LNP testing, colloidal LNP dispersions were adjusted to an mRNA concentration of 5–20 ng μL^{-1} using ribonucleases free water for dilution. 10 µL of the respective diluted samples were added to the cells equaling an amount of 50 – 200 ng mRNA per well in a total volume of 100 µL. The cells were further incubated for 24 h at 37 °C and 5% CO_2. On day 3, cell viability was determined using CellTiter 96® AQueous Non-Radioactive Cell Proliferation Assay (MTS) according to manufacturer's protocol (Promega GmbH). The absorbance signal (at 400 nm) was quantified with a multiplate reader (Infinite® M200 PRO, Tecan, Männedorf, Switzerland).

1.8. Luciferase assay

Luciferase assay was conducted with several immortal cell lines: HeLa, Jurkat, C2C12, HepG2, A549. Cell lines were grown according to standard cell culture conditions.

Transfection and readout with adherent cells: One day before transfection 10,000 cells per well were seeded into a 96 well plate in a volume of 100 µL of the respective medium (containing 10% FBS) and cultured for 24 h at 37 °C and 5% CO_2. On day 2, old medium was removed and 90 µL of minimal essential medium (MEM) (Gibco, Art. Nr. 51200038) was added to the cells. LNP nanodispersions were adjusted to an mRNA concentration of 10 ng μL^{-1} using ribonucleases free water for dilution. 10 µL of the respective diluted samples were added to the cells equaling an amount of 100 ng mRNA per well in a total volume of 100 µL. After 4 h, old medium containing residual samples was removed and replaced with 100 µL of fresh medium (containing 10% FBS). Cells were further incubated for 20 h at 37 °C and 5% CO_2. On day 3, transfection efficiency was determined using OneGlo™ Luciferase Assay System (Promega GmbH, Walldorf, Germany). The luminescence signal was quantified with the Infinite® M200 PRO multiplate reader.

Transfection and readout with suspension cells: On the day of transfection, 50,000 cells per well were seeded into a 96-well plate in a volume of 90 µL of the respective medium (containing 10% FBS). Samples were adjusted to an mRNA concentration of 10 ng μL^{-1} using ribonucleases free water for dilution. 10 µL of the respective diluted samples were added to the cells equaling an amount of 100 ng mRNA per well in a total volume of 100 µL. Cells were further incubated for 24 h at 37 °C and 5% CO_2. On day 2, transfection efficiency was determined using OneGlo™ Luciferase Assay System (Promega GmbH). The luminescence signal was quantified with the Infinite® M200 PRO multiplate reader.

For all transfection experiments, jetMESSENGER® (Polyplus®) was used as positive control. Reagents were prepared according to manufacturer's protocols and applied at an equal RNA dose per well as the test samples.

1.9. Ribogreen assay

Colloidal LNP dispersions were characterized by RiboGreen Assay. The Thermo Fischer Quant-iT™ RiboGreen™ RNA Assay Kit was used. The procedure was performed according to manufacturer's protocol with slight adjustments. Samples were diluted to a theoretical RNA concentration of 0.4 µg mL^{-1} using either TE buffer or Triton buffer and added to a 96 well plate at a volume of 100 µL. For dissolution of LNPs in the presence of Triton-buffer the plate was placed into an incubator for 10 min at 37 °C and 5% CO_2. 100 µL dye solution were added to each well followed by thorough pipetting. Fluorescence signals were measured with the Infinite® 200 PRO microplate reader at an excitation/emission value of 480/520 nm. All samples and standards were measured in duplicates.

1.10. Agarose gel electrophoresis (AGE)

Colloidal LNP dispersions were characterized by agarose gel electrophoresis. The assay was carried out using the E-Gel™ power snap electrophoresis system by Thermo Fisher Scientific. For the evaluation of the samples a 1% agarose gel with a volume capacity of 20 µL per well was used. Agarose gel electrophoresis demonstrates full encapsulation of mRNA for all tested LNP compositions (within the detection range of SYBR Safe staining).

1.11. Cell Microscopy

Light microscopy images were taken on a Microscopic Axio Observer Z1 from Zeiss.

2. Experimental Procedures

2.1. Synthesis of 1-chloro-3-methoxy-propan-2-ol

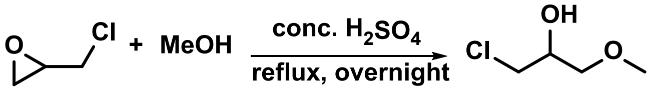

A 1 L three-necked flask equipped with neodym stir bar, reflux condenser and dropping funnel was charged with MeOH (306 g, 388 mL, 9.57 mol) and sulfuric acid (98%, 7 mL, 125 mmol). The flask was immersed in an oil bath and epichlorohydrin (295 g, 250 mL, 3.19 mol) was added dropwise through the dropping funnel to the solution under vigorous stirring. After complete addition of epichlorohydrin, the reaction was heated to reflux and stirred overnight under reflux. The reaction mixture was cooled to room temperature and $BaCO_3$ (37.0 g, 187 mmol) was added under vigorous stirring. After 1 h of stirring, excess MeOH was evaporated under reduced pressure. 1-Chloro-3-methoxy-propan-2-ol (320 g, 2.60 mol, 81%) was obtained as colorless liquid after fractional distillation (T_b = 83 – 87 °C, p = 85 mbar) of the residue.

^{1}H NMR (400 MHz, DMSO-*d*$_6$) δ [ppm]: 5.26 (d, J = 5.3 Hz, 1H), 3.87 – 3.71 (m, 1H), 3.61 (dd, J = 11.0, 4.5 Hz, 1H), 3.51 (dd, J = 11.0, 5.6 Hz, 1H), 3.33 (dd, 2H), 3.26 (s, 3H).

^{13}C NMR (100 MHz, DMSO-*d*$_6$) δ [ppm]: 73.53 (CH_2), 68.93 (CH), 58.51 (CH_3), 47.12 (CH_2).

2.2. Synthesis of glycidyl methyl ether (GME)

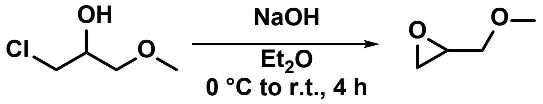

1-Chloro-3-methoxy-propan-2-ol (320 g, 2.57 mol) was added to a three-necked flask equipped with mechanical stirrer, thermometer and reflux condenser. Diethyl ether (320 mL) was added and the solution was cooled to 0 °C with an ice bath under stirring. Sodium hydroxide (123 g, 3.08 mol) was added portion wise to the solution under vigorous stirring while the temperature was kept below 15 °C. The reaction mixture was allowed to reach room temperature over 4 h. The reaction mixture was filtered through a G2 frit and the filter was washed four times with diethyl ether (160 mL). The combined organic phases were dried over $MgSO_4$ (96 g) and filtered through a pleated filter. The filter

was washed with diethyl ether (160 mL). Diethyl ether was evaporated under reduced pressure. Glycidyl methyl ether (156 g, 1.77 mol, 69%) was obtained as a colorless liquid after fractional distillation (T_b = 72–74 °C, p = 300 mbar) of the residue.
^{1}H NMR (400 MHz, DMSO-*d*$_6$) δ [ppm]: 3.63 (dd, J = 11.4, 2.6 Hz, 1H), 3.28 (s, 3H), 3.16 (dd, J = 11.4, 6.5 Hz, 1H), 3.12 – 3.04 (m, 1H), 2.72 (dd, J = 5.1, 4.2 Hz, 1H), 2.53 (dd, J = 5.1, 2.7 Hz, 1H).
^{13}C NMR (100 MHz, DMSO-*d*$_6$) δ [ppm]: 72.90 (CH_2), 58.27 (CH_3), 50.14 (CH), 43.27 (CH_2).

2.3. Synthesis of 1-methoxy-3-(2-methoxyethoxy)propan-2-ol (MMEPOH)

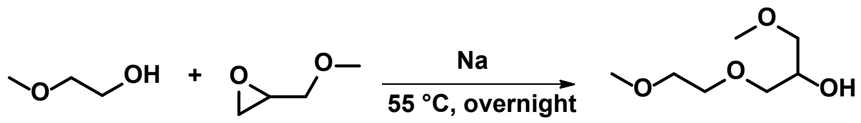

A flame-dried two-necked Schlenk flask equipped with stir bar, septum and reflux condenser was charged with 2-methoxy ethanol (33.6 g, 36.0 mL, 442 mmol) under argon. The flask was immersed in an ice-bath. Sodium (2.84 g, 124 mmol) was added portion wise to the alcohol under stirring and argon. The reaction mixture was allowed to reach room temperature. After complete reaction of the sodium, the solution was heated to 55 °C and GME (9.31 g, 9.50 mL, 106 mmol) was slowly added via syringe pump (1 mL h^{-1}) under argon. The reaction mixture was stirred overnight, cooled to room temperature and neutralized by the addition of aqueous 2 M HCl solution. After evaporation of water and excess 2-methoxy ethanol under reduced pressure, 1-methoxy-3-(2-methoxyethoxy)propan-2-ol (12.03 g, 73.2 mmol, 69%) was obtained after fraction distillation (T_b = 60 °C, $p = 5\cdot10^{-2}$ mbar) of the residue.
^{1}H NMR (400 MHz, DMSO-*d*$_6$) δ [ppm]: 4.79 (d, J = 5.2 Hz, 1H), 3.76 – 3.60 (m, 1H), 3.54 – 3.46 (m, 2H), 3.46 – 3.39 (m, 2H), 3.39 – 3.16 (m, 10H).
^{13}C NMR (100 MHz, DMSO-*d*$_6$) δ [ppm]: 74.26 (CH_2), 72.63 (CH_2), 71.28 (CH_2), 69.92 (CH_2), 68.38 (CH), 58.41 (CH_3), 58.09 (CH_3).

2.4. Synthesis of MeO-P(EO-*co*-GME)-OH (rPEG)

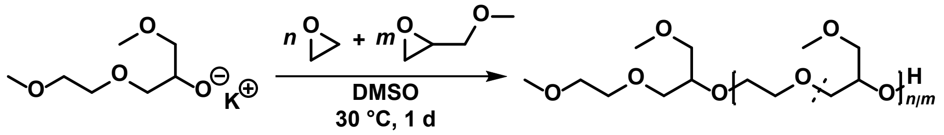

Caveat: Ethylene oxide is a highly flammable and toxic gas. It must be handled by trained researchers and staff!
Potassium *tert*-butoxide (KO*t*Bu) (233 mg, 2.08 mmol) was dissolved in stabilizer-free THF and small quantities of Millipore water and transferred into a flame-dried and argon flushed flask equipped with a Teflon stopcock and a septum. MMEPOH (348 mg, 2.12 mmol) was dissolved in benzene and transferred into the flask. High vacuum was applied to the flask and the solvents were removed under high vacuum at 30 °C. The resulting initiator salt was further dried under high vacuum at 55 °C overnight. The initiator salt was dissolved in dry DMSO (30 mL). After freezing the resulting solution at -80 °C, CaH_2-dried glycidyl methyl ether (GME) (3.92 g, 4.00 mL, 44.5 mmol) was added to the flask via syringe. Ethylene oxide (EO) (1.94 g, 2.00 mL, 44.5 mmol) was added to the flask via cryo-transfer from a graduated ampoule. The cooling bath was removed, and the reaction mixture was allowed to reach room temperature. The resulting solution was stirred for 1 d at 30 °C under static high vacuum. After full conversion (reaction control via ^{1}H NMR spectroscopy) of the monomers, the flask was ventilated and DMSO was evaporated under reduced pressure. The residue was redissolved in diethyl ether (74 mL) and toluene (9 mL) and acetic acid (380 mg, 360 µL, 6.36 mmol) was added under stirring. After 15 min, the reaction mixture was filtered through a dense layer of celite. MeO-P(EO-*co*-GME)-OH (6.15 g, 95%) was obtained as a yellow-colored viscous liquid after evaporation of residual solvents and excess acetic acid under reduced pressure.
^{1}H NMR (400 MHz, $CDCl_3$) δ [ppm]: 3.85-3.36 (m, polyether backbone), 3.34 (s, OCH_3).

2.5. Synthesis of rPEG lipids

2.5.1. Synthesis of MeO-P(EO-*co*-GME)-COOH

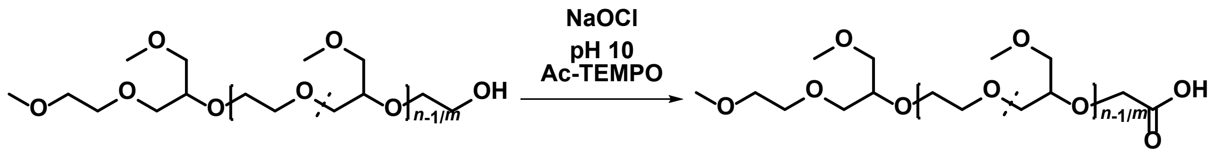

MeO-P(EO-*co*-GME)-OH (7 g, 3.5 mmol) was charged to a round bottom flask. Water (42 mL), sodium carbonate (Na_2CO_3, 1 M, 7 mL), sodium bicarbonate ($NaHCO_3$, 1M, 7 mL), sodium bromide (NaBr, 0.1M, 3.5 mL) and 4-acetamido-2,2,6,6-tetramethylpiperidine-1-oxyl (Ac-TEMPO, 37.3 mg) were added and the reaction mixture was cooled to 0 – 5 °C. Then, bleach (7.5 wt%, 7.2 mL) was added in 4 portions within 1 h and stirred for 1 additional h. Sodium thiosulphate ($Na_2S_2O_3$, 1M, 3.5 mL) was added to quench the reaction. Then, DCM (35 mL) was added, and the pH was adjusted by adding HCl (1M) to pH = 1 – 3. Organic product phase was separated, and water phase was extracted with DCM (35 mL). DCM-phases were combined and dried over night with $MgSO_4$. After filtration, the organic solvent was removed under vacuum to yield the crude MeO-P(EO-*co*-GME)-COOH (7.6 g yield) as an oil. MeO-P(EO-*co*-GME-COOH was utilized in the next step without further purification

2.5.2.Synthesis of MeO-P(EO-*co*-GME)-*N,N*-ditetradecylacetamide (rPEG-DTAA)

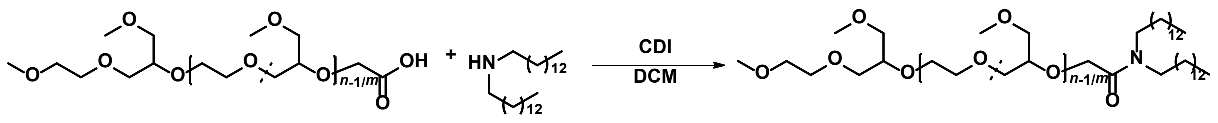

Crude MeO-P(EO-*co*-GME)-COOH (7 g, 3.5 mmol) was dissolved in DCM (70 mL) at room temperature. Carbonyl diimidazole (680 mg, 4.2 mmol) was added to the reaction solution and the reaction mixture was stirred at room temperature for 1 – 2 h. Then *N,N*-ditetradecylacetamine (1 g, 3.5 mmol) was added to the reaction and the reaction mixture was stirred at room temperature for additional 16 h. Conversion was quantified by high-performance liquid chromatography (HPLC) equipped with a charged aerosol detector (CAD) in area% (%a). Water was added to the reaction mixture and the biphasic system was stirred for 30 min before phases were separated. Additional DCM (70 mL) was added to the water phase for an additional extraction. The organic phase was concentrated before the product was purified by chromatography. Then, the product containing fractions were pooled and the solvent was removed under vacuum. The desired product MeO-P(EO-*co*-GME)-*N,N*-ditetradecylacetamide (rPEG-DTAA) was isolated in 29% yield. Purity was determined by HPLC (99.3%a).

^{1}H NMR (400 MHz, $CDCl_3$) *δ* [ppm]: 4.37 – 4.16 (m, 2H) 3.84–3.34 (m, polyether backbone), 3.33 (s, OCH_3), 3.30 – 3.15 (m, 4H), 1.57 – 1.43 (m, 4H), 1.31 – 1.16 (m, 44H) 0.86 (t, *J* = 6.8 Hz, 6H).

^{13}C NMR (100 MHz, $CDCl_3$) *δ* [ppm]: 169.10 (N-C=O), 78.72–78.31 (CH_2), 72.88–72.61 (CH_2), 71.31–69.99 (CH_2), 59.32 (CH_3), 32.01 (CH_2), 29.77–29.40 (CH_2), 29.00 (CH_2), 27.65–26.98 (CH_2), 14.22 (CH_3).

2.5.3.Synthesis of MeO-P(EO-*co*-GME)-OTs

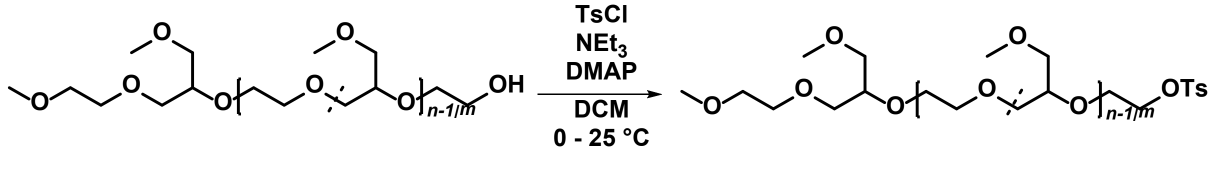

A 50 mL round bottom flask was charged with MeO-P(EO-*co*-GME)-OH (2.46 g, 910 µmol) and DCM (2.46 mL) at room temperature. The solution was cooled to 0–5 °C under stirring. Subsequently, DMAP (11.2 mg, 91.7 µmol), NEt_3 (166 mg, 1.64 mmol) and *p*-toluenesulfonyl chloride (TsCl) (263 mg, 1.37

mmol) were added successively. The reaction mixture was stirred at 0–5 °C for another 1 h, then heated to 20–25 °C and stirred for 72 h. Conversion was quantified by HPLC. Due to incomplete conversion of the starting material NEt_3 (55.3 mg, 546 µmol) and TsCl (87.7 mg, 460 µmol) were added at 20–25 °C and stirring was continued for another 18 h. Then, DCM (22.1 mL) and water (14.8 mL) were added, and the biphasic mixture was stirred vigorously for 5 min. The phases were separated, and the organic layer was mixed with water (4.92 mL) and 1 N HCl solution (0.54 mL). After stirring for 10 min the phases were separated and the organic layer was washed with water (4.92 mL). The organic phase was dried over anhydrous Na_2SO_4, filtered, and concentrated on a rotary evaporator with a water bath temperature of 60 °C at a pressure of 700–5 mbar. MeO-P(EO-*co*-GME)-OTs was isolated as an orange oil (2.14 g, 749 µmol, 82.3% yield) and utilized in the next step without further purification.

2.5.4.Synthesis of MeO-P(EO-*co*-GME)-DHG (dihydroxy glycerol)

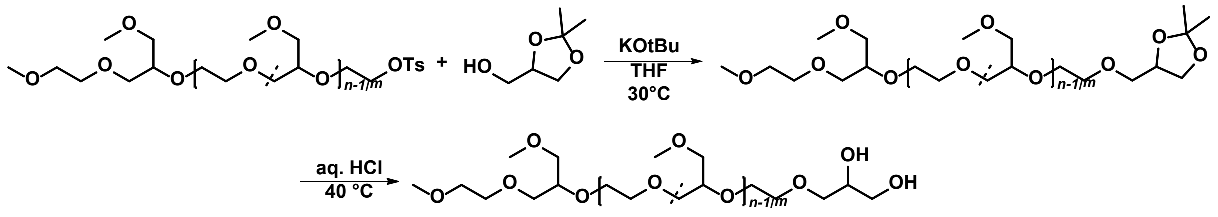

A 50 mL round bottom flask was charged with MeO-P(EO-*co*-GME)-OTs (1.00 g, 350 µmol, 88.7%a purity), anhydrous THF (4 mL) and heated to 30 °C. In a separate glass vessel solketal (92.6 mg, 700 µmol) and KO*t*Bu (78.6 mg, 700 µmol) were mixed in anhydrous THF (3 mL). The orange suspension was added in portions to the MeO-P(EO-*co*-GME)-OTs solution and stirring was continued at 30 °C for 6 h. Water (4 mL) was added and THF was removed on a rotary evaporator with a water bath temperature of 60 °C at a pressure of 400–150 mbar. The remaining aqueous solution was extracted with DCM (8 mL) and the organic layer was concentrated on a rotary evaporator with a water bath temperature of 60 °C at a pressure of 700–20 mbar. The intermediate MeO-P(EO-*co*-GME)-IPG (0.63 g) was isolated as an orange oil.

Subsequently, the MeO-P(EO-*co*-GME)-IPG intermediate was dissolved in water (6.30 mL) and the pH was adjusted to 1.5–1.6 using 0.1N HCl solution (227 µL). The yellow solution was heated to 40 °C for 75 min, cooled to room temperature and extracted with DCM (2×12.6 mL). The organic phases were combined and concentrated on a rotary evaporator with a water bath temperature of 60 °C at a pressure of 700–5 mbar. MeO-P(EO-*co*-GME)-DHG (430 mg, 160 µmol) was isolated as an orange oil.

2.5.5.Synthesis of MeO-P(EO-*co*-GME)-DMG (rPEG-DMG)

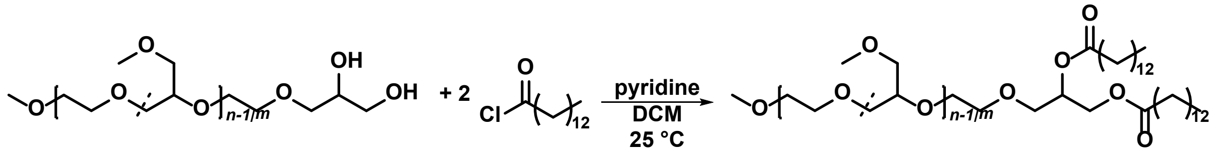

A 250 mL round bottom flask was charged with MeO-P(EO-*co*-GME)-DHG (12.0 g, 3.66 mmol) and DCM (60 mL). The solution was stirred for 5 min at 25 °C. Py (2.17 g, 27.5 mmol) and MyCl (2.71 g; 11.0 mmol) were added successively and the reaction mixture was stirred at 25 °C for 24 h. DCM (60 mL) and water (48 mL) were added to the crude product mixture, and the pH was adjusted to approx. 1.5 using 1M HCl solution (36 mL). The DCM phase was separated and the organic solvent was removed on a rotary evaporator with a water bath temperature of 60 °C at a pressure of 700–5 mbar to yield crude MeO-P(EO_{26}-*co*-GME_{23})-DMG as an oil. Purification was carried out by flash column chromatography on a Buchi (Essen, Germany) Pure C-850 FlashPrep system using a 330 g Zeochem Silica Materials (Quebec City, Canada) SiliaSep PREMIUM Flash C18 cartridge (25 µm), a ACN to *i*PrOH gradient and a flow rate of 50 mL/min. Product containing fractions were combined and concentrated.

MeO-P(EO-*co*-GME)-DMG (rPEG-DMG, 6.20 g, 1.68 mmol, 48%) was isolated as a pale yellow oil. Purity was determined by HPLC (purity 99%a).

^{1}H NMR (400 MHz, $CDCl_3$) δ [ppm]: 5.19–5.14 (m, 1H), 4.30 (dd, J = 12.0, 3.5 Hz, 1H), 4.11 (dd, J = 11.9, 6.5 Hz, 1H), 3.84–3.33 (m, polyether backbone), 3.31 (s, OCH_3), 2.27 (t, J = 7.5 Hz, 2H), 2.25 (t, J = 7.6 Hz, 2H), 1.60–1.52 (m, 4H), 1.29–1.19 (m, 40H), 0.84 (t, J = 6.8 Hz).

^{13}C NMR (100 MHz, $CDCl_3$) δ [ppm]: 173.30 (O-C=O), 78.72–78.24 (CH_2), 72.78–69.54 (CH_2), 62.74 (CH_2), 59.24 (CH_3), 34.26 (CH_2), 31.96 (CH_2), 29.72–29.13 (CH_2), 24.98 (CH_2), 22.72 (CH_2), 14.17 (CH_3).

3. Additional Figures

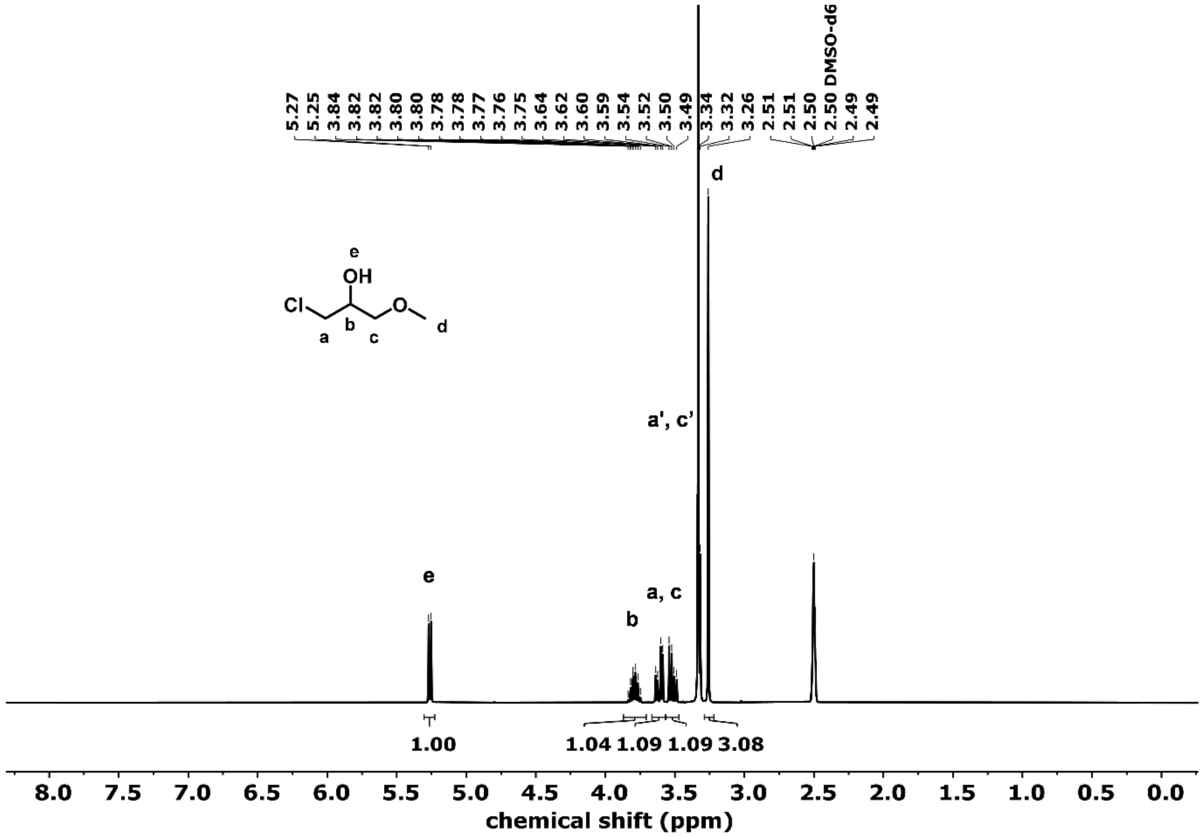

Figure S1. ^{1}H NMR spectrum (DMSO-d_6, 400 MHz) of 1-chloro-3-methoxy-propan-2-ol; Integration of signal at 3.33 (dd) is not possible because of resonance from water traces in DMSO-d_6.

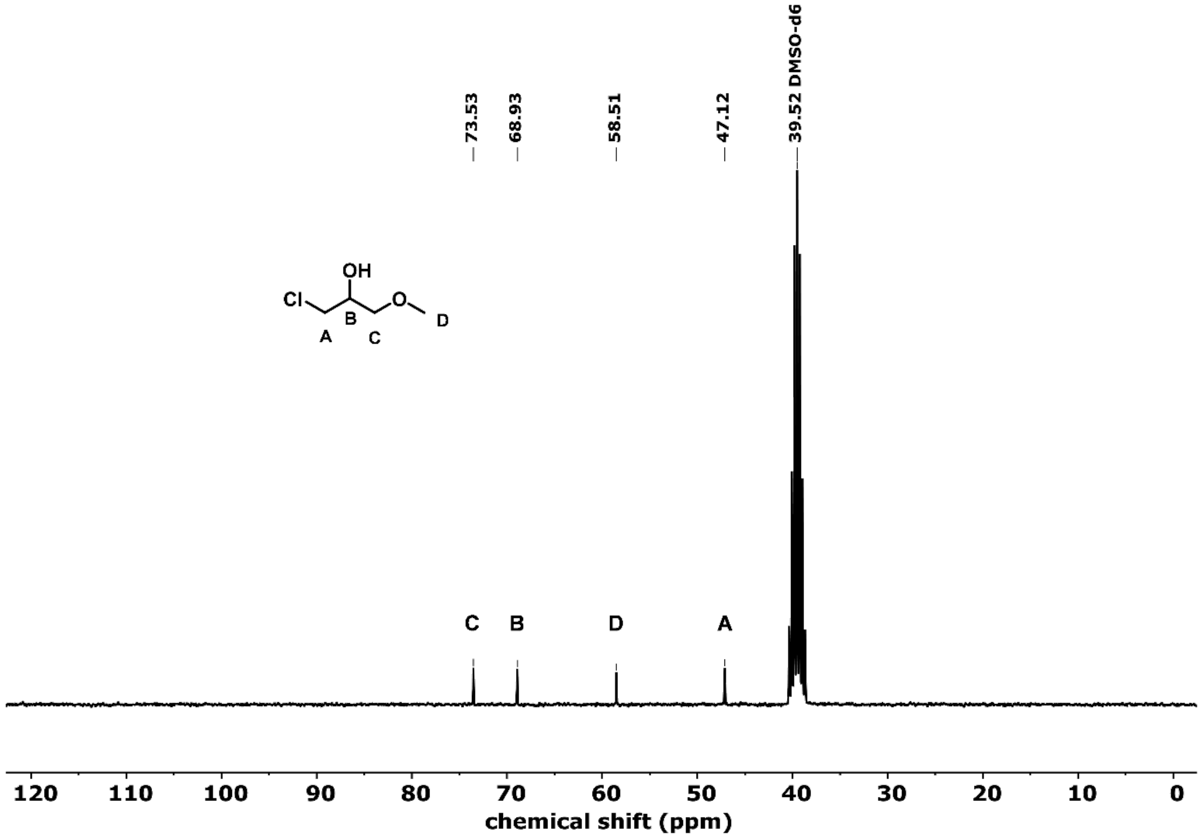

Figure S2. ^{13}C NMR spectrum (DMSO-d_6, 100 MHz) of 1-chloro-3-methoxy-propan-2-ol.

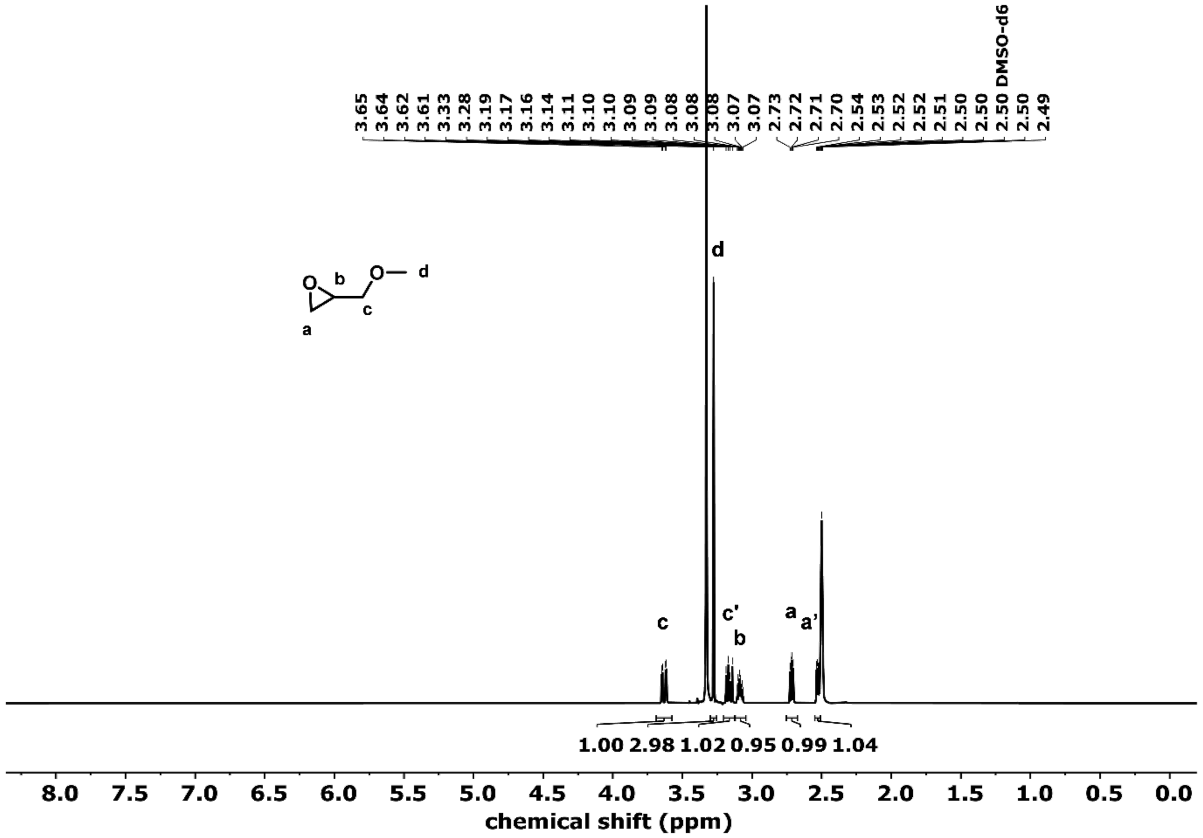

Figure S3. ^{1}H NMR spectrum (DMSO-d_6, 400 MHz) of glycidyl methyl ether.

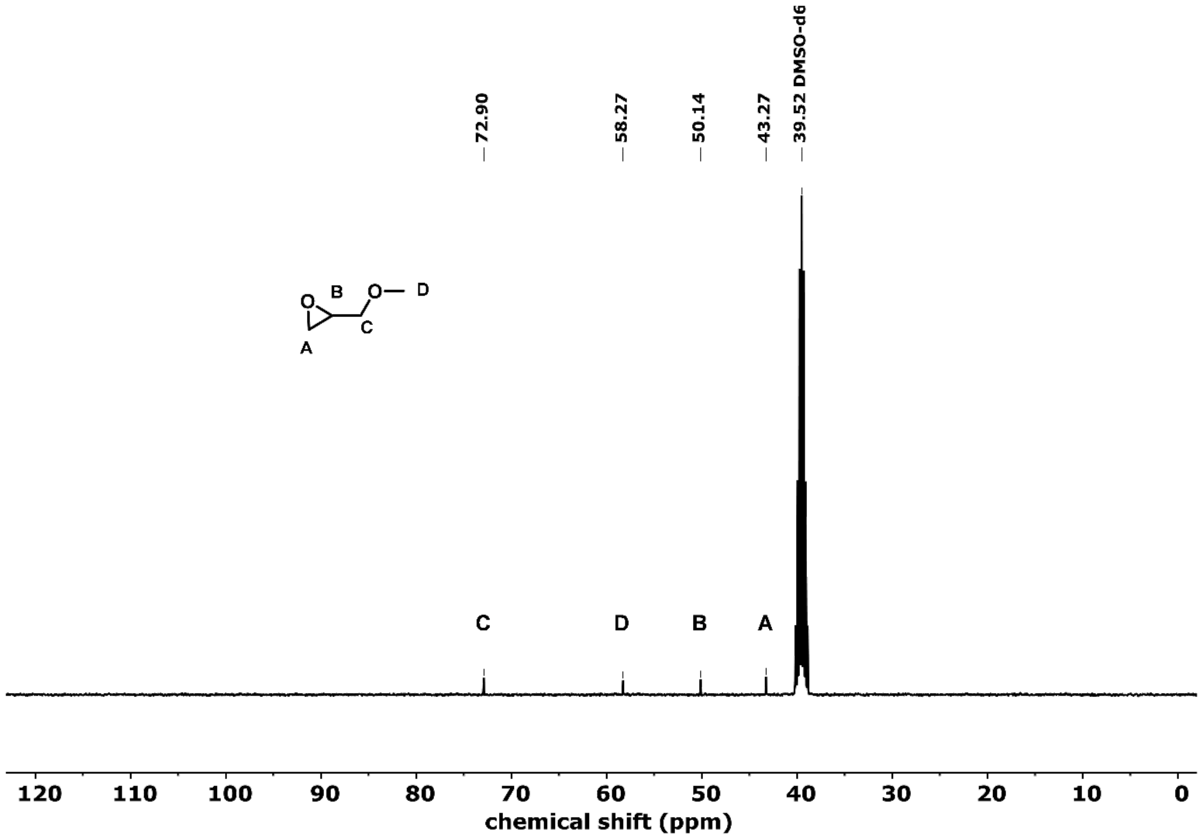

Figure S4. ^{13}C NMR spectrum (DMSO-d_6, 100 MHz) of glycidyl methyl ether.

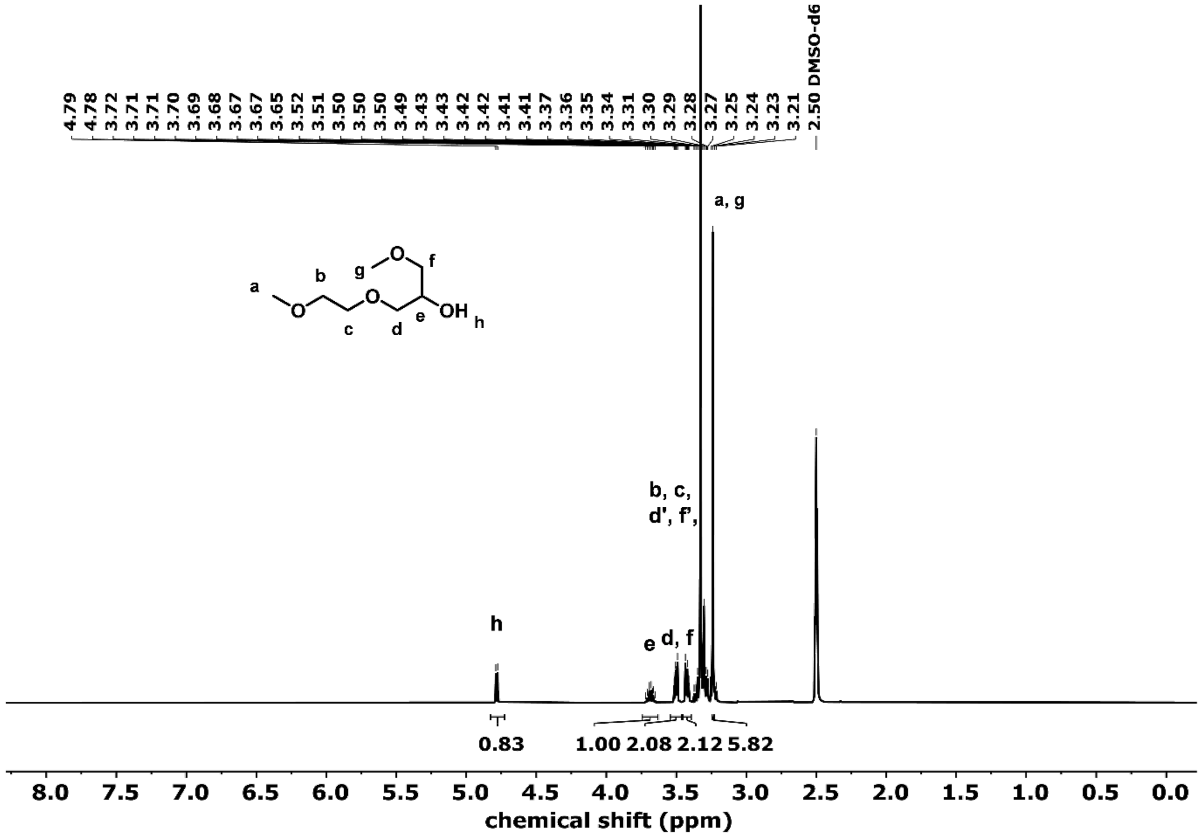

Figure S5. ^{1}H NMR spectrum (DMSO-d_6, 400 MHz) of 1-methoxy-3-(2-methoxyethoxy)propan-2-ol; Integration of signal at 3.39 – 3.16 (m) is not possible because of resonance from water traces (3.33 ppm) in DMSO-d_6.

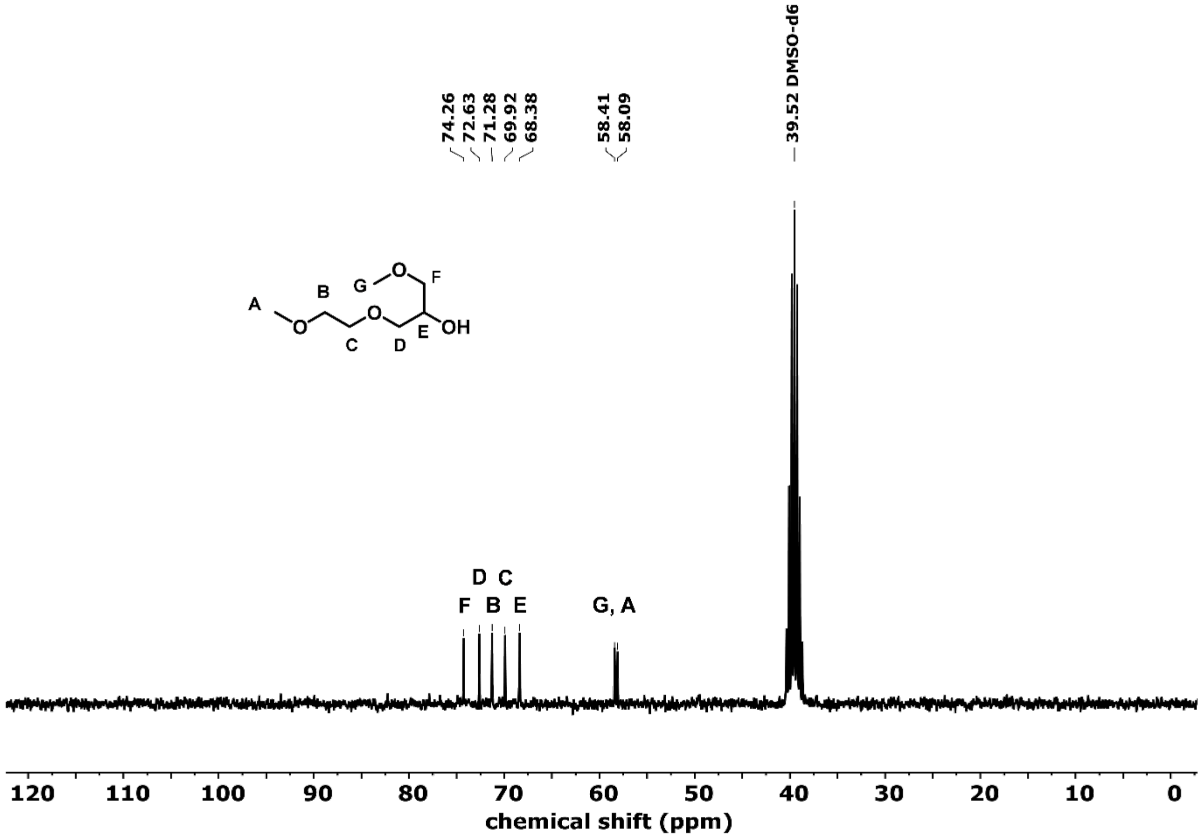

Figure S6. ^{13}C NMR spectrum (DMSO-d_6, 100 MHz) of 1-methoxy-3-(2-methoxyethoxy)propan-2-ol.

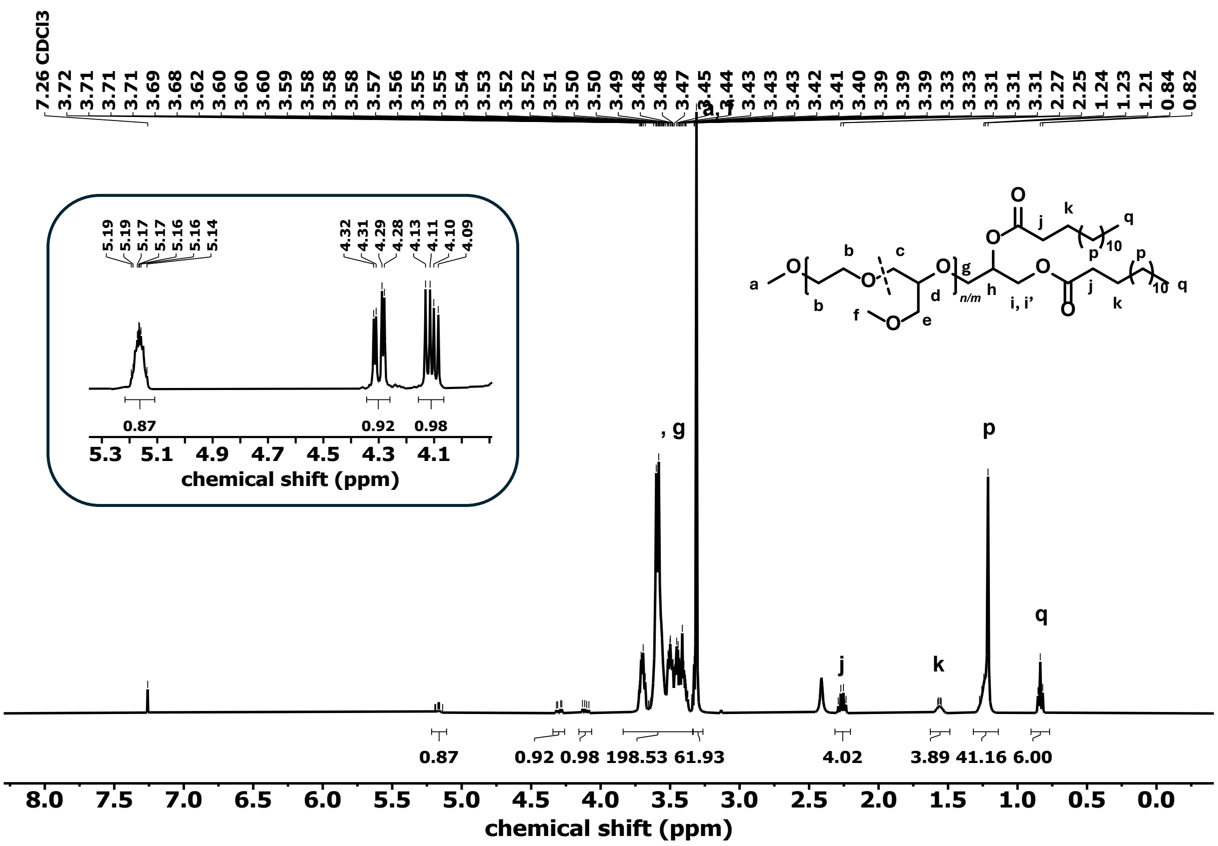

Figure S7. Exemplary ^{1}H NMR spectrum (CDCl$_3$, 400 MHz) of rPEG$_{44}^{0.48}$-DMG.

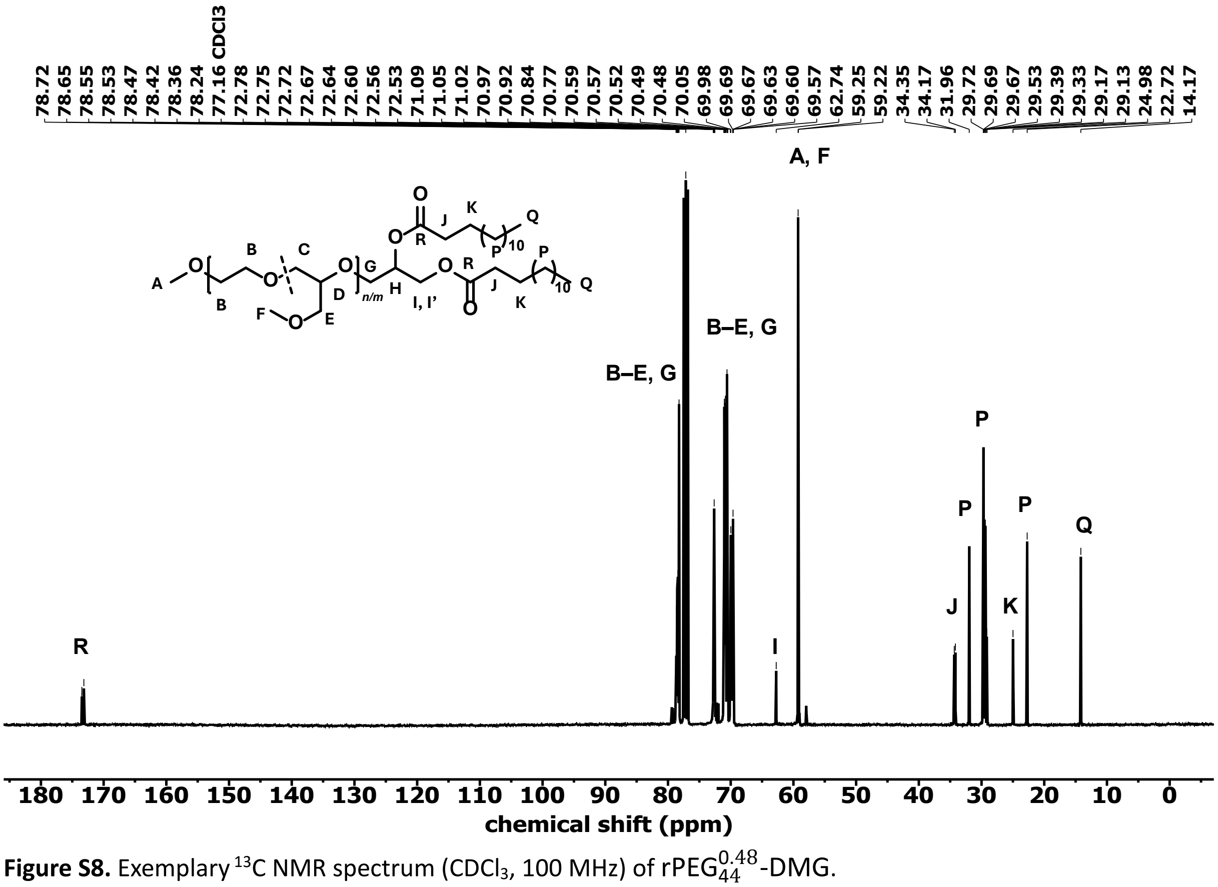

Figure S8. Exemplary ^{13}C NMR spectrum (CDCl$_3$, 100 MHz) of rPEG$_{44}^{0.48}$-DMG.

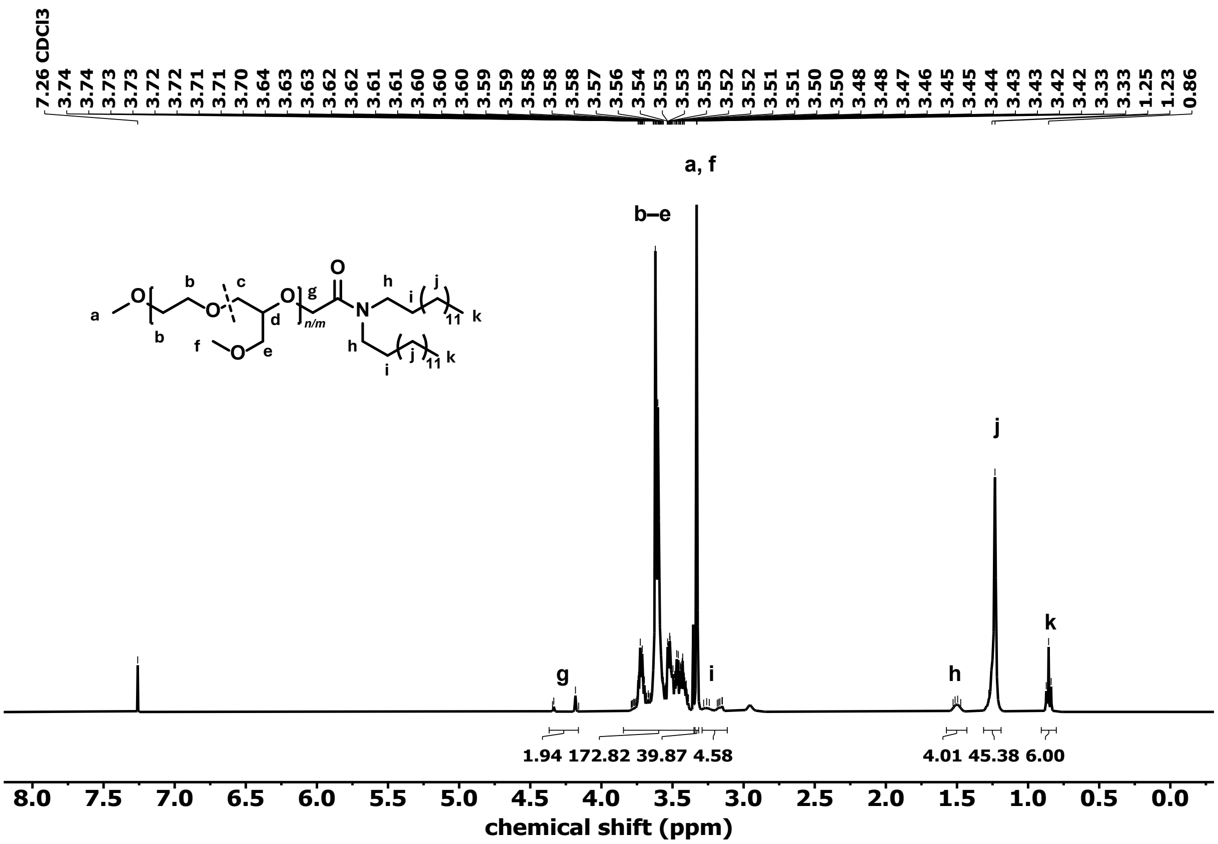

Figure S9. Exemplary ^{1}H NMR spectrum ($CDCl_3$, 400 MHz) of rPEG$_{43}^{0.36}$-DTAA.

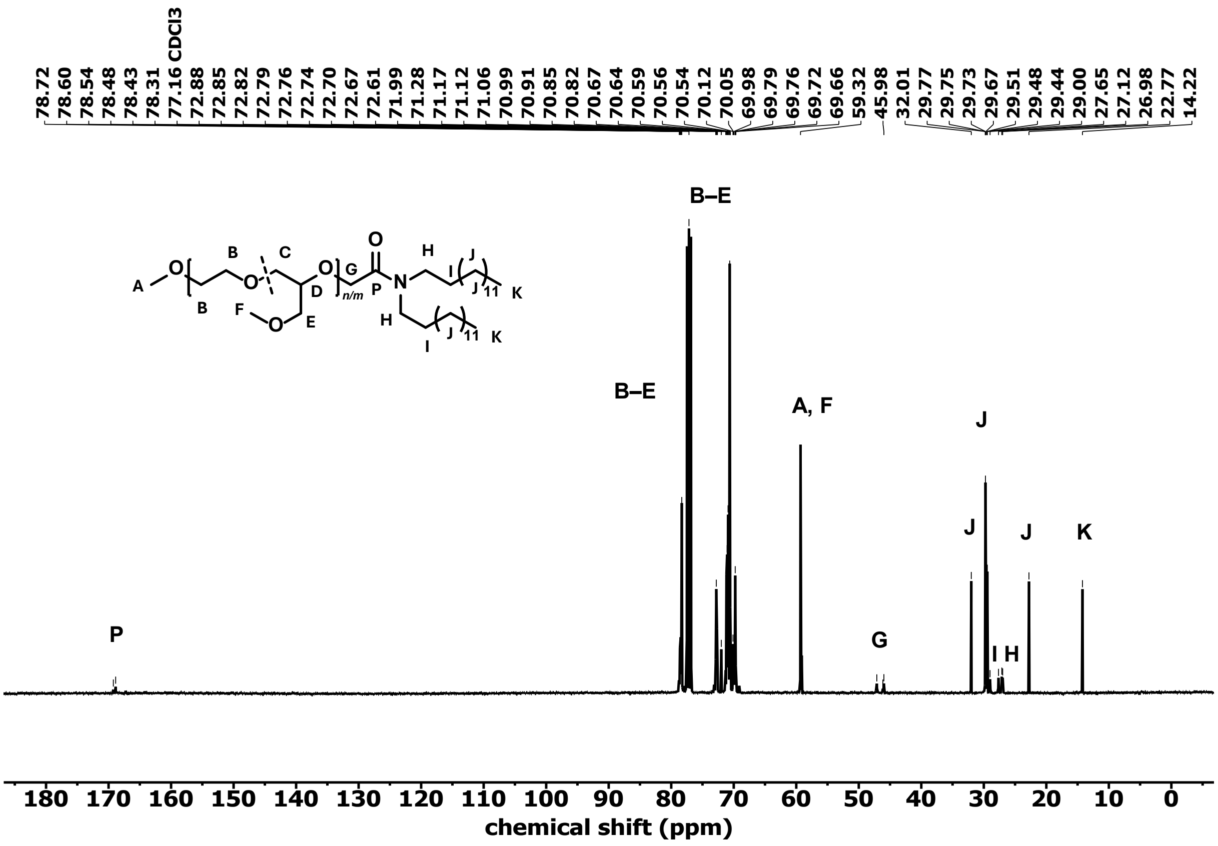

Figure S10. Exemplary ^{13}C NMR spectrum ($CDCl_3$, 100 MHz) of rPEG$_{43}^{0.36}$-DTAA.

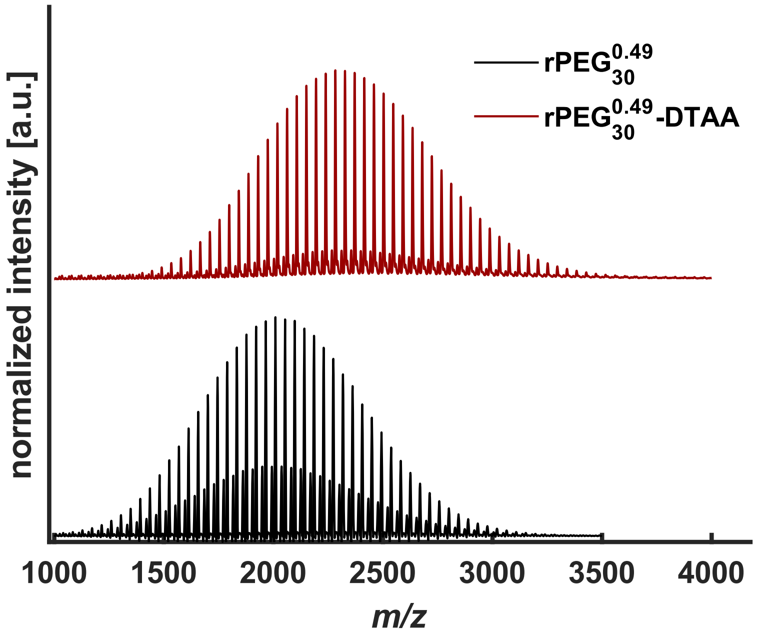

Figure S11. Stacked MALDI-ToF mass spectra of synthesized rPEG$^{0.49}_{30}$ and rPEG$^{0.49}_{30}$-DTAA.

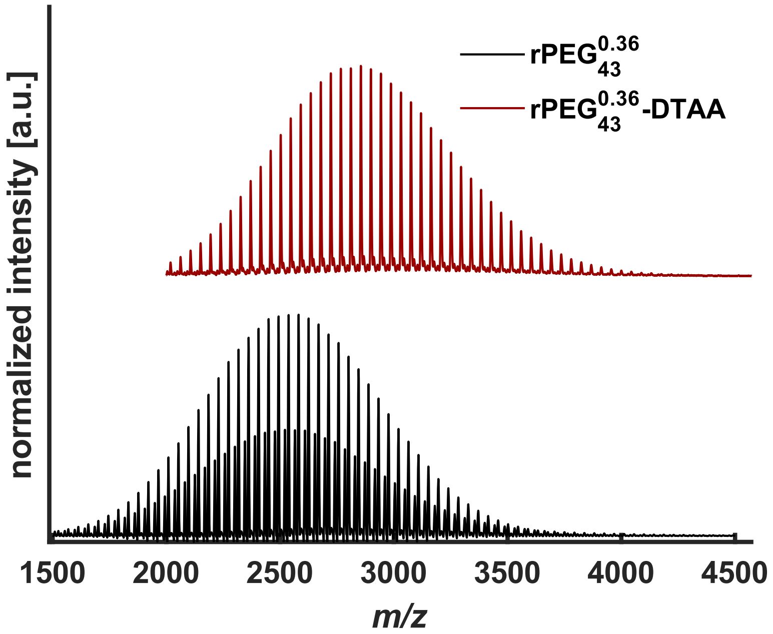

Figure S12. Stacked MALDI-ToF mass spectra of rPEG$^{0.36}_{43}$ and rPEG$^{0.36}_{43}$-DTAA.

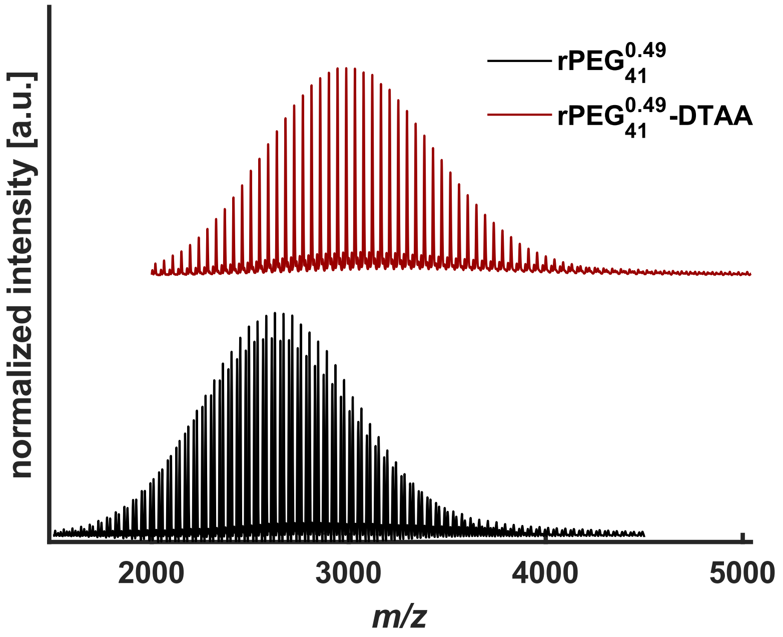

Figure S13. Stacked MALDI-ToF mass spectra of rPEG$_{41}^{0.49}$ and rPEG$_{41}^{0.49}$-DTAA.

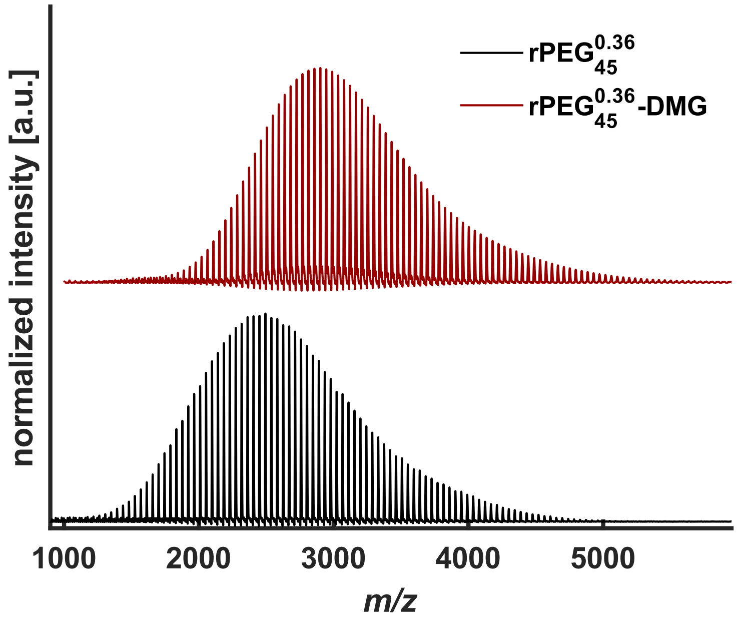

Figure S14. Stacked MALDI-ToF mass spectra of rPEG$_{45}^{0.36}$ and rPEG$_{45}^{0.36}$-DMG.

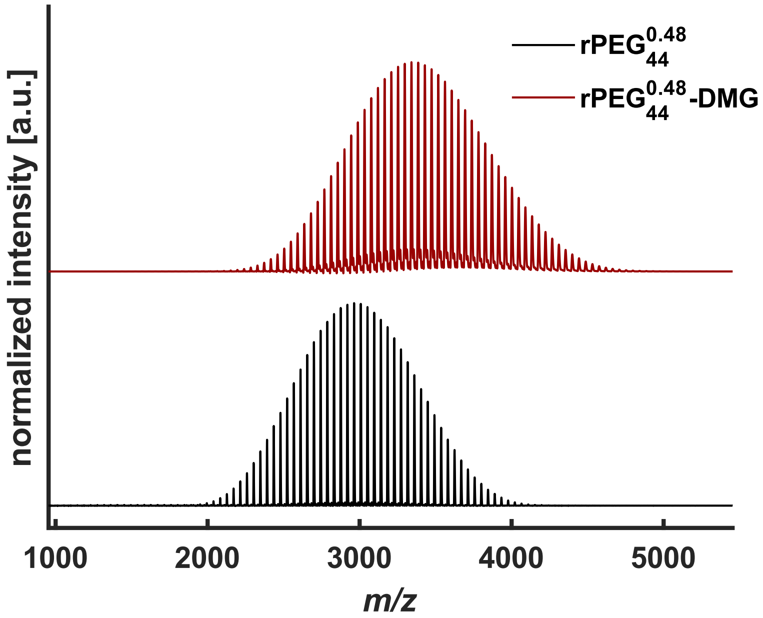

Figure S15. Stacked MALDI-ToF mass spectra of rPEG$_{44}^{0.48}$ and rPEG$_{44}^{0.48}$-DMG.

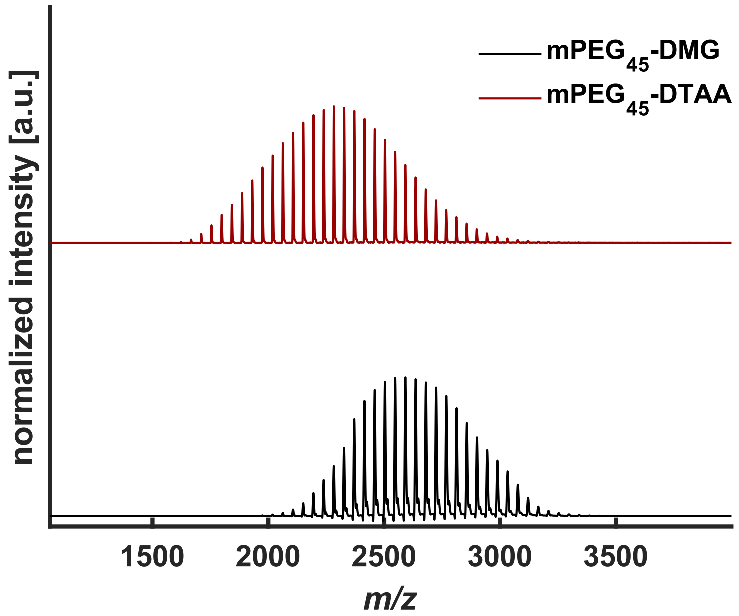

Figure S16. Stacked MALDI-ToF mass spectra of mPEG-DMG and mPEG-DTAA.

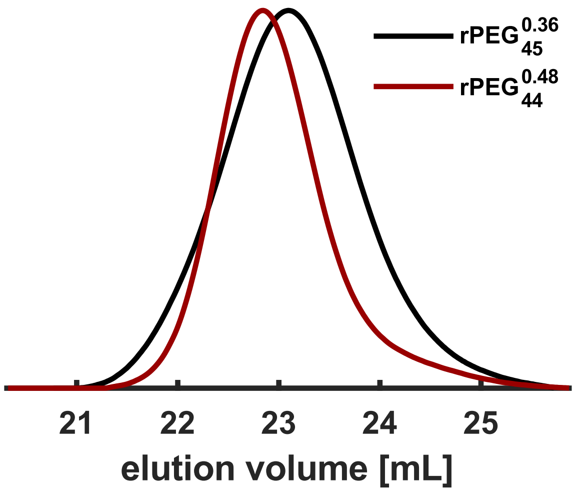

Figure S17. Stacked SEC traces of rPEG precursors for rPEG-DMG.

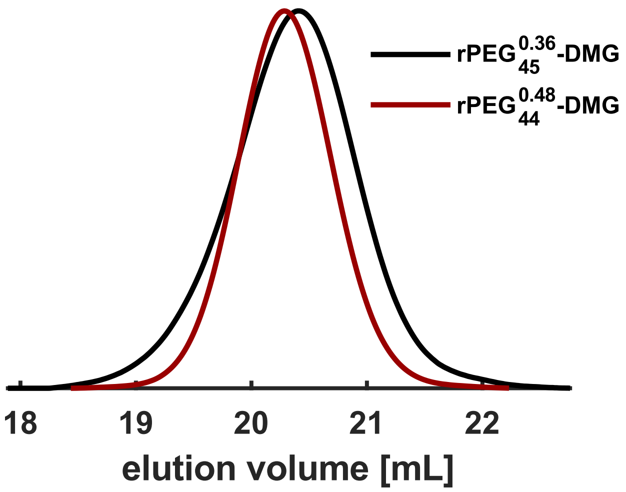

Figure S18. Stacked SEC traces of rPEG-DMGs.

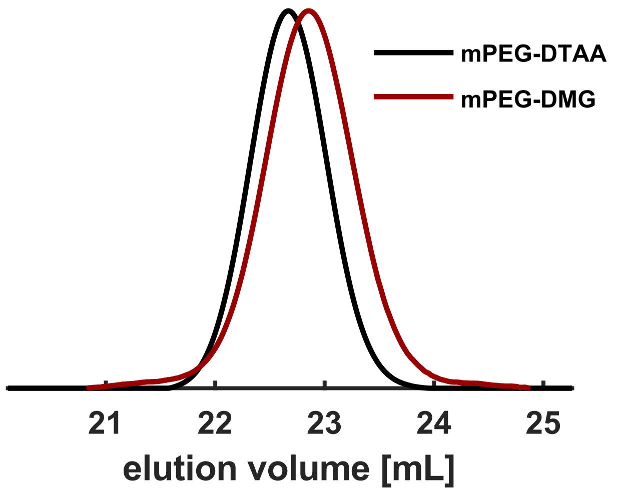

Figure S19. Stacked SEC traces of mPEG lipids.

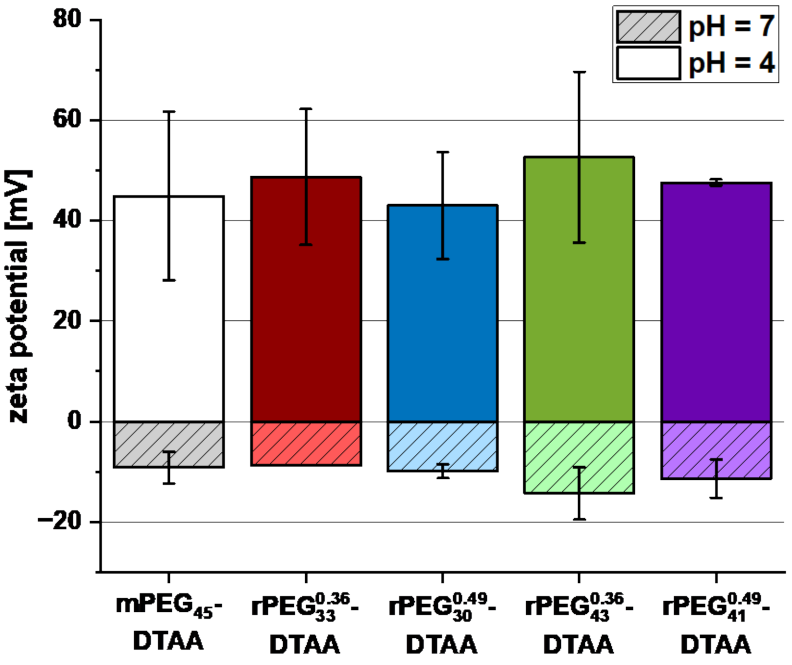

Figure S20. Zeta potentials of mPEG$_{45}$-DTAA and rPEG-DTAA LNPs.

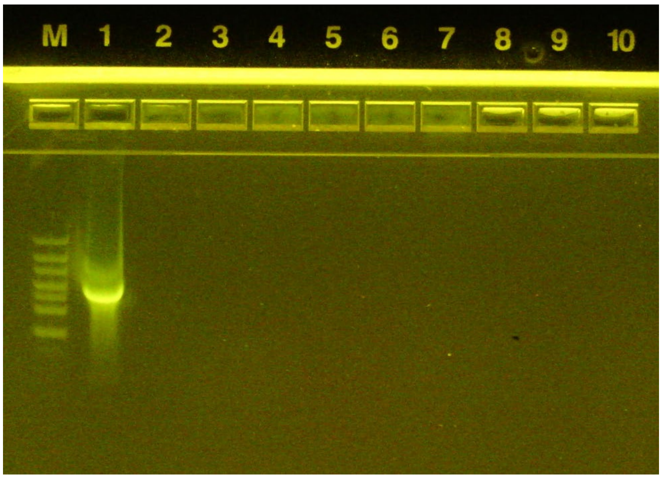

M	RNA Ladder
1	800 ng FLuc mRNA
2	mPEG$_{45}$-DMG
3	mPEG$_{45}$-DTAA
4	rPEG$^{0.49}_{30}$-DTAA
5	rPEG$^{0.36}_{33}$-DTAA
6	rPEG$^{0.49}_{41}$-DTAA
7	rPEG$^{0.36}_{43}$-DTAA

Figure S21. AGE of mPEG$_{45}$-DMG, mPEG$_{45}$-DTAA and rPEG-DTAA LNPs.

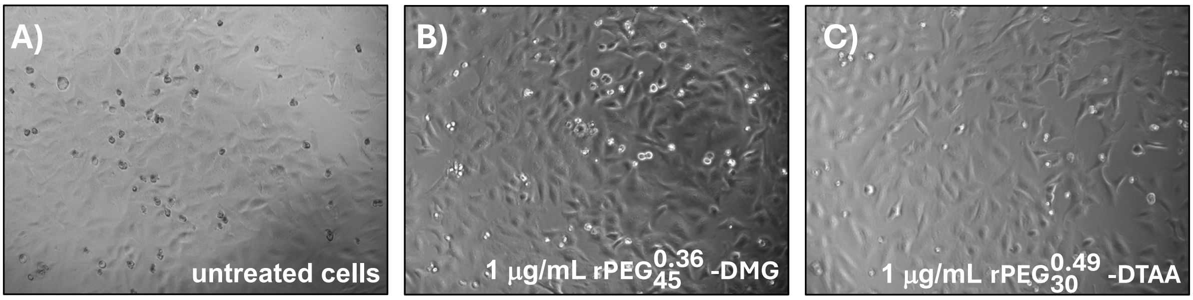

Figure S22. Microscopy images of (A) untreated cells, (B) cells treated with rPEG$^{0.36}_{45}$-DMG, (C) cells treated with rPEG$^{0.49}_{30}$-DTAA.

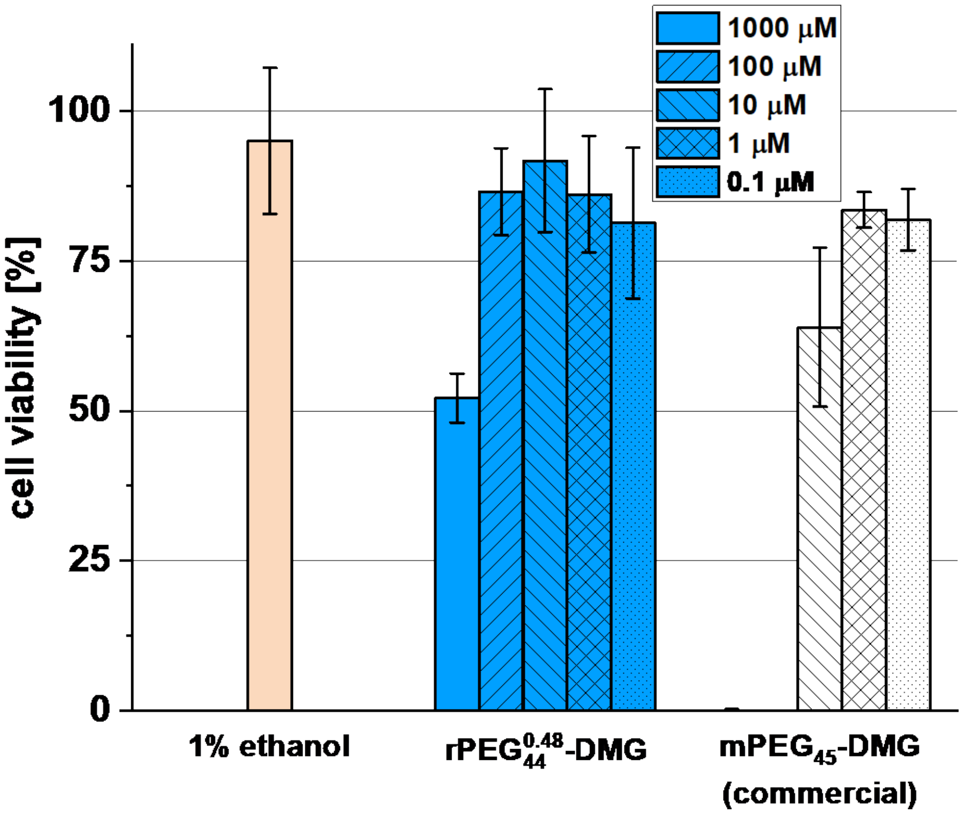

Figure S23. MTS assay of mPEG$_{45}$-DMG and rPEG$^{0.48}_{44}$-DMG with HeLa cells.

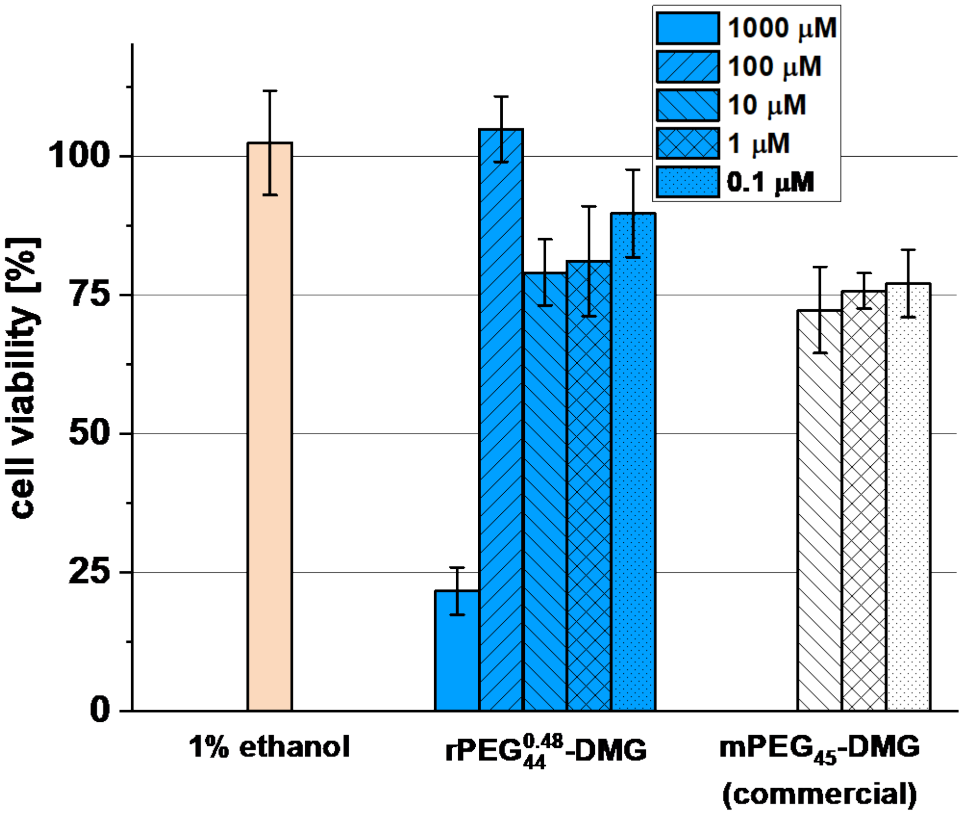

Figure S24. MTS assay of mPEG$_{45}$-DMG and rPEG$^{0.48}_{44}$-DMG with HepG2 cells.

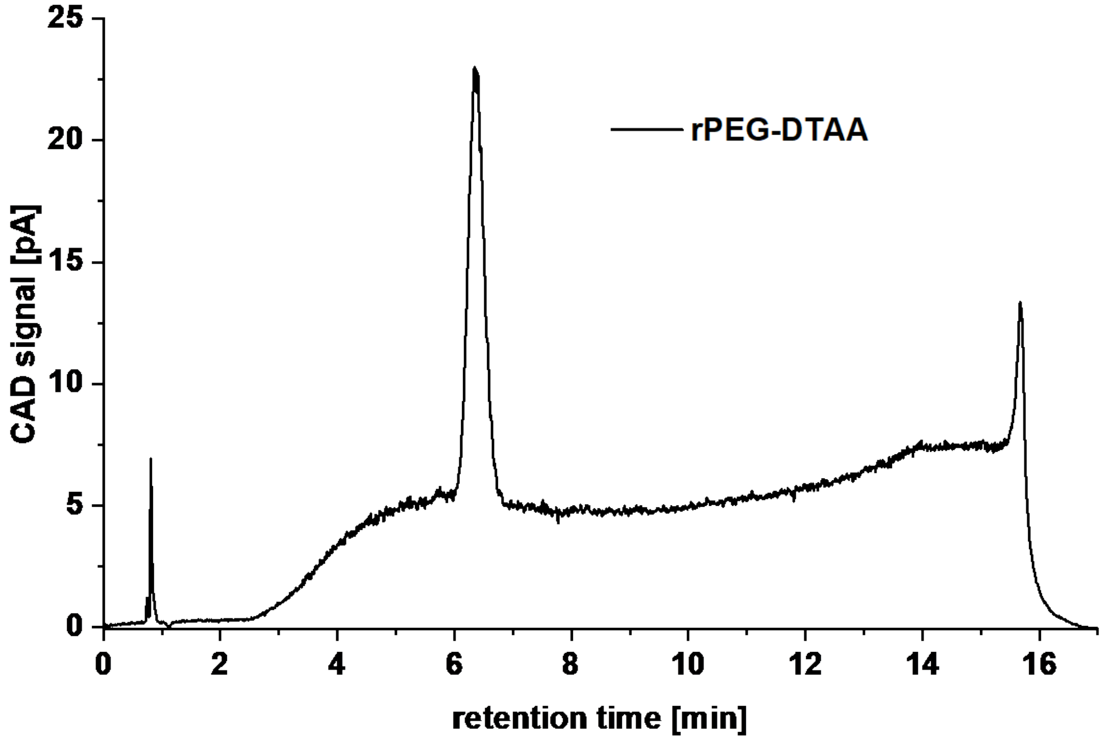

Figure S25. Exemplary HPLC elugram of rPEG-DTAA.

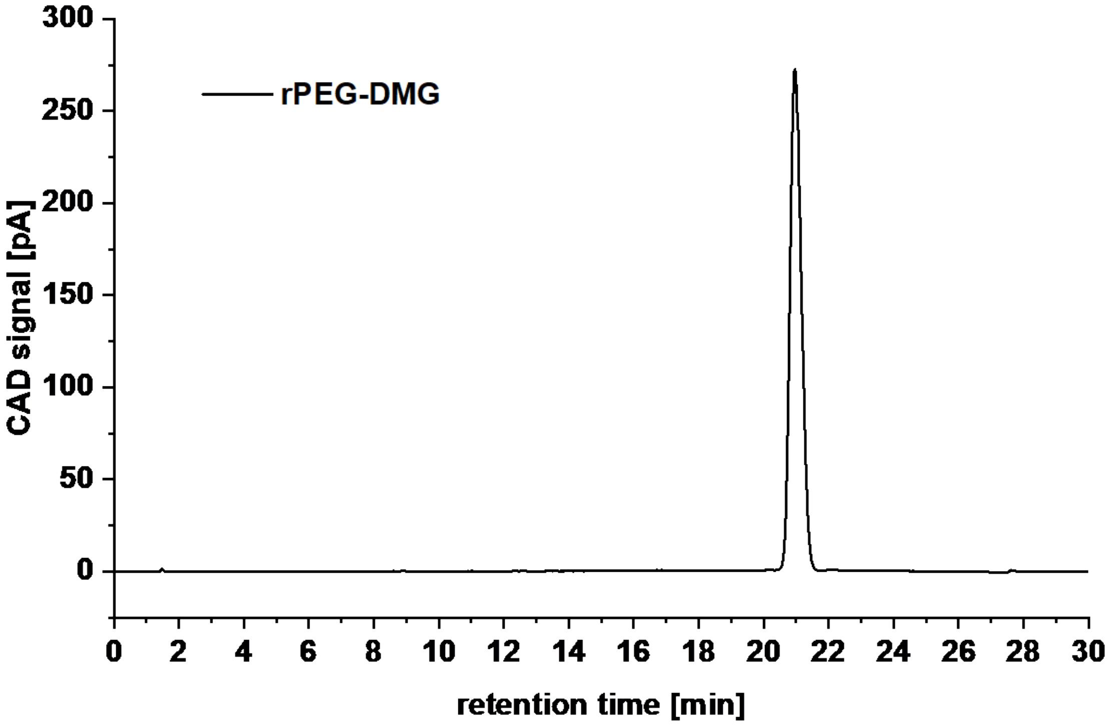

Figure S26. Exemplary HPLC elugram of rPEG-DMG.

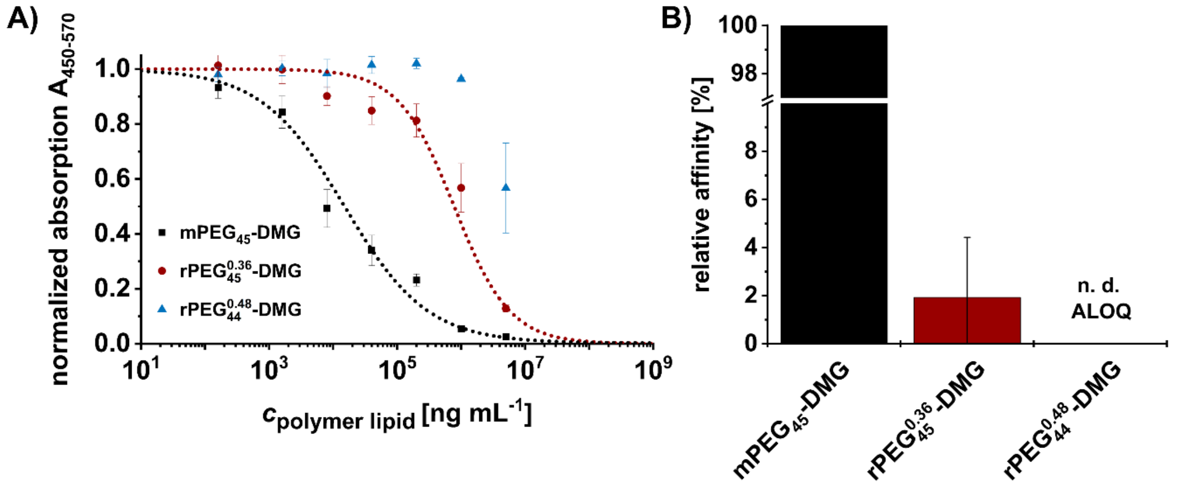

Figure S27. Interaction of mPEG and rPEG lipids and backbone-specific APA. (A) ELISA of mPEG- and rPEG-DMG; (B) relative APA affinities normalized to the EC_{50} of mPEG-DMG. The relative affinity of $rPEG_{44}^{0.48}$-DMG is not determined (n. d.) because its EC_{50} is above the limit of quantification (ALOQ).

4. Additional Tables

Table S1. Overview of characterization of synthesized rPEG.

**Sample**	$DP_{calc.}$	$DP_{MALDI+NMR}$	**GME$_{calc}$ [%]**	**GME$_{NMR}$ [%]**	**$M_{n,calc.}$ [kg mol^{-1}]**	**$M_{n,MALDI}$ [kg mol^{-1}]**	**$M_{n,SEC}$^{a} [kg mol^{-1}]**	**$Đ_{SEC}$^{a}**
$rPEG_{33}^{0.36}$	33	33	36	36	2.0	2.0	1.6	1.05
$rPEG_{30}^{0.49}$	30	30	50	49	2.0	2.1	1.6	1.04
$rPEG_{43}^{0.36}$	44	43	36	36	2.7	2.6	2.1	1.05
$rPEG_{41}^{0.49}$	44	41	50	49	2.9	2.7	2.1	1.06
$rPEG_{45}^{0.36}$	45	45	36	36	2.8	2.6	2.0	1.10
$rPEG_{44}^{0.48}$	44	44	50	48	3.1	3.0	2.2	1.08

^{a}Eluent: DMF (with 1 mg mL^{-1} anhydrous LiBr), PEG calibration, RI detector.

Table S2. Characterization data of MeO-P(EO-*co*-GME)-*N,N*-ditetradecylacetamide compounds prepared.

**sample**	**conversion by HPLC [%a]**
$rPEG_{33}^{0.36}$-DTAA	59
$rPEG_{30}^{0.49}$-DTAA	26
$rPEG_{43}^{0.36}$-DTAA	47
$rPEG_{41}^{0.49}$-DTAA	36
$mPEG_{45}$-DTAA	90

Table S3. Size and zeta potential of LNPs.

**sample**	**z-ave. [nm]**	**PDI**	**z-pot. (before dialysis) [mV]**	**z-pot. (after dialysis) [mV]**
$rPEG_{33}^{0.36}$-DTAA	122.8±16.8	0.175±0.030	48.7±13.5	-8.8±0.0
$rPEG_{30}^{0.49}$-DTAA	137.1±8.8	0.166±0.040	43.0±10.7	-9.9±1.4
$rPEG_{43}^{0.36}$-DTAA	110.2±13.0	0.151±0.030	52.6±17.0	-14.3±5.2
$rPEG_{41}^{0.49}$-DTAA	111.0±10.5	0.134±0.052	47.6±0.6	-11.4±3.8
$mPEG_{45}$-DTAA	109.3±6.5	0.172±0.071	44.9±16.8	-9.2±3.2
$rPEG_{45}^{0.36}$-DMG	104.2±1.4	0.169±0.005	62.8	-0.154
$mPEG_{45}$-DMG	102.9±2.7	0.219±0.003	69.5	11.1

Table S4. MTS assay results for mPEGs and rPEGs.

sample	cell viability [%]					
	**1000 µg mL^{-1}**	**100 µg mL^{-1}**	**10 µg mL^{-1}**	**1 µg mL^{-1}**	**0.1 µg mL^{-1}**	**0.01 µg mL^{-1}**
$rPEG_{33}^{0.36}$	108±4	107±6	107±4	108±4	110±1	102±2
$rPEG_{30}^{0.49}$	72±6	90±9	102±12	101±7	100±11	103±4
$rPEG_{43}^{0.36}$	112±4	107±2	106±4	106±1	109±3	101±3
$rPEG_{41}^{0.49}$	60±14	85±11	104±6	103±5	106±8	103±2
$mPEG_{45}$	103±2	102±6	103±5	109±12	102±7	96±3
Untreated Cells	100	100	100	100	100	100

Table S5. MTS assay results for commercial mPEG-DMG and rPEG-DMG with HeLa cells.

sample	cell viability [%]				
	**1000 µM**	**100 µM**	**10 µM**	**1 µM**	**0.1 µM**
$mPEG_{45}$-DMG	0.006±0.29	-0.45±0.13	64±13	84±3	82±5
$rPEG_{44}^{0.48}$-DMG	52±4	87±7	92±12	86±10	81±13

Table S6. MTS assay results for commercial mPEG-DMG and rPEG-DMG with HepG2 cells.

sample	cell viability [%]				
	1000 µM	100 µM	10 µM	1 µM	0.1 µM
$mPEG_{45}$-DMG	-11±0.51	-11±0.25	72±8	76±3	77±6
$rPEG_{44}^{0.48}$-DMG	22±4	105±6	79±6	81±10	90±8

Table S7. MTS assay results for mPEG-DTAA and rPEG-DTAA LNPs.

sample	c(mRNA) [µg mL^{-1}]		
	**2**	**1**	**0.5**
	**cell viability [%]**		
$rPEG_{33}^{0.36}$-DTAA	96±2	96±2	102±2
$rPEG_{30}^{0.49}$-DTAA	42±2	89±5	102±2
$rPEG_{43}^{0.36}$-DTAA	98±2	101±1	102±1
$rPEG_{41}^{0.49}$-DTAA	102±2	99±2	101±2
$mPEG_{45}$-DTAA	99±2	99±2	100±1
$rPEG_{45}^{0.36}$-DMG	100±2	94±1	95±2
$mPEG_{45}$-DMG	88±1	97±2	95±2
Untreated Cells	100	100	100

Table S8. Ribogreen assay results for LNPs.

sample	RNA encapsulation [%]
$rPEG_{33}^{0.36}$-DTAA	90.1
$rPEG_{30}^{0.49}$-DTAA	89.9
$rPEG_{43}^{0.36}$-DTAA	83.4
$rPEG_{41}^{0.49}$-DTAA	89.2
$mPEG_{45}$-DTAA	91.4
$rPEG_{45}^{0.36}$-DMG	55.9
$mPEG_{45}$-DMG	56.8

Table S9. Competetive ELISA results for $mPEG_{45}$-DTAA and rPEG-DTAA.

sample	EC_{50} [ng ml^{-1}]	relative affinity [%]
$mPEG_{45}$-DTAA	$1.6 \cdot 10^4 \pm 3 \cdot 10^4$	100
$rPEG_{33}^{0.36}$-DTAA	ALOQ[a)]	n. d./ALOQ[b)]
$rPEG_{30}^{0.49}$-DTAA	ALOQ[a)]	n. d./ALOQ[b)]
$rPEG_{43}^{0.36}$-DTAA	$1.6 \cdot 10^6 \pm 1.5 \cdot 10^6$	0.96 ± 0.88
$rPEG_{41}^{0.49}$-DTAA	ALOQ[a)]	n. d./ALOQ[b)]

[a)]Above the concentration limit; reported as above the limit of quantification (ALOQ).

[b)]Effectively close to zero but reported as not determined (n.d.) since their EC_{50} values were ALOQ.

Table S10. Competetive ELISA results for $mPEG_{45}$-DMG and rPEG-DMG.

sample	EC_{50} [ng ml^{-1}]	relative affinity [%]
$mPEG_{45}$-DMG	$1.5 \cdot 10^4 \pm 8 \cdot 10^4$	100
$rPEG_{45}^{0.36}$-DMG	$7.8 \cdot 10^5 \pm 9 \cdot 10^5$	1.9 ± 2.5
$rPEG_{44}^{0.48}$-DMG	ALOQ[a)]	n. d./ALOQ[b)]

[a)]Above the concentration limit; reported as above the limit of quantification (ALOQ).

[b)]Effectively close to zero but reported as not determined (n.d.) since their EC_{50} values were ALOQ.

5. References

1 J. Schmidt, A.-L. Ziegler, F. T. Kaps, L. J. Rosenberger, M. Bros, H. Frey and R. Luxenhofer, *Macromol. Rapid Commun.,* 2025, e00781

2 B.-M. Chen, Y.-C. Su, C.-J. Chang, P.-A. Burnouf, K.-H. Chuang, C.-H. Chen, T.-L. Cheng, Y.-T. Chen, J.-Y. Wu, and S. R. Roffler, *Anal. Chem.*, 2016, **88**, 10661

3 E. Schneck, I. Berts, A. Halperin, J. Daillant and G. Fragneto, *Biomaterials*, 2015, **46**, 95–104.

4 J. Pusterla, E. Scoppola, C. Appel, T. Mukhina, C. Shen, G. Brezesinski and E. Schneck, *Nanoscale*, 2022, **14**, 15048–15059.

5 L. G. Parratt, *Phys. Rev.*, 1954, **95**, 359–369.

6 A. Baer, I. Hoffmann, N. Mahmoudi, A. Poulhazan, M. J. Harrington, G. Mayer, S. Schmidt and E. Schneck, *Small*, DOI:10.1002/smll.202300516.
